# Zeolitic imidazolate frameworks activate endosomal Toll-like receptors and potentiate immunogenicity of SARS-CoV-2 spike protein trimer

**DOI:** 10.1126/sciadv.adj6380

**Published:** 2024-03-06

**Authors:** Shahad K. Alsaiari, Seba Nadeef, John L. Daristotle, William Rothwell, Bujie Du, Johnny Garcia, Linzixuan Zhang, Morteza Sarmadi, Timothy A. Forster, Nandita Menon, Stacey Qiaohui Lin, Lisa H. Tostanoski, Nicole Hachmann, Erika Yan Wang, John D. Ventura, Dan H. Barouch, Robert Langer, Ana Jaklenec

**Affiliations:** ^1^ David H. Koch Institute for Integrative Cancer Research, Massachusetts Institute of Technology, Cambridge, MA 02139, USA.; ^2^ Department of Pathology, Massachusetts General Hospital, Boston, MA 02114, USA.; ^3^ Department of Chemical Engineering, Massachusetts Institute of Technology, Cambridge, MA 02139, USA.; ^4^ Center for Virology and Vaccine Research, Beth Israel Deaconess Medical Center, Harvard Medical School, Boston, MA 02215, USA.

## Abstract

Nanomaterials offer unique opportunities to engineer immunomodulatory activity. In this work, we report the Toll-like receptor agonist activity of a nanoscale adjuvant zeolitic imidazolate framework–8 (ZIF-8). The accumulation of ZIF-8 in endosomes and the pH-responsive release of its subunits enable selective engagement with endosomal Toll-like receptors, minimizing the risk of off-target activation. The intrinsic adjuvant properties of ZIF-8, along with the efficient delivery and biomimetic presentation of a severe acute respiratory syndrome coronavirus 2 spike protein receptor-binding domain trimer, primed rapid humoral and cell-mediated immunity in a dose-sparing manner. Our study offers insights for next-generation adjuvants that can potentially impact future vaccine development.

## Teaser

ZIF-8 is an immunostimulatory vehicle capable of selectively activating endosomal TLRs upon cargo release.

## INTRODUCTION

Subunit protein antigens are safer and easier to manufacture than traditional whole pathogen vaccines (*1*–*3*), but they often require coadministration with an adjuvant to potentiate antigen presentation and produce effective immune outcomes (*4*, *5*). Rationally designed vaccine adjuvants aim to trigger specific receptors on antigen-presenting cells (APCs) to direct the immune system to respond in a T helper 1, T helper 2, or T helper 17 manner (*6*, *7*). Pattern recognition receptors (PRRs), particularly Toll-like receptors (TLRs), expressed mainly on APCs play a critical role in the modulation of these immune responses (*8*–*10*). PRR agonists help generate greater immune response magnitude and long-lasting memory response against vaccine targets when coadministered with subunit protein antigens (*11*, *12*). Nevertheless, only a few PRR adjuvants are licensed for use in U.S. Food and Drug Administration–approved vaccines owing to safety and tolerability concerns. However, these concerns can be potentially mitigated by novel delivery approaches including nanoparticle (NP)–based methods (*13*, *14*).

Biomimetic NP delivery strategies have been shown to enhance antibody titers even at ultralow antigen doses (*4*). A range of NPs have demonstrated adjuvant activity through enhancing the codelivery of antigens and adjuvants to APCs and lymph nodes (LNs) (*4*, *15*). NPs allow for passive vaccine drainage to LNs (*12*, *14*), create multivalent antigen interactions with B cells, and facilitate antigen presentation onto class I major histocompatibility complex (MHC I) molecules that enhance CD8^+^ T cell responses (*16*). Several studies have revealed that polymeric NP antigens (*17*, *18*) and liposomes (*19*) activate stimulatory immune pathways such as TLRs or inflammasomes in the absence of adjuvants and antigens. The intrinsic immune characteristics of NPs should be explored to avoid potential alteration in immune signal processing by APCs that result from the excessive activation of innate immune responses. Investigating NPs’ immunostimulatory properties offers an opportunity to study novel carriers that not only tune the spatiotemporal distribution of vaccine antigen but also directly engage with innate signaling pathways and enable selective activation of immunologic pathways.

Zeolitic imidazolate framework-8 (ZIF-8)—a subclass of metal organic frameworks—encapsulates antigen through a biomimetic mineralization process, affording exceptional protection from biological, thermal, and chemical degradation while maintaining bioavailability (*20*–*22*). ZIF-8 is also established as an efficient vaccine delivery platform (*23*–*25*). Preclinical studies demonstrated that ZIF-8 modulates vaccine-induced immune responses and stimulates high antibody responses through the codelivery of antigen and adjuvant to APCs (*23*, *24*). However, the contribution of ZIF-8’s composition to its adjuvant properties is poorly characterized. Imidazole, the basic building block of ZIF-8, is the smallest moiety in synthetic TLR agonists and can be modified to produce TLR-specific agonist or antagonist molecules, especially TLR-7 and TLR-8 (*26*, *27*). Here, we investigated how ZIF-8 NPs can modulate innate immune activation to enhance adaptive immune responses to protein antigens, using severe acute respiratory syndrome coronavirus 2 (SARS-CoV-2) spike protein receptor-binding domain (RBD) trimer as a model antigen. To better understand how ZIF-8 improves immunogenicity, we used biodistribution studies, immunological techniques, and transcriptome sequencing.

We found that ZIF-8 can passively drain to the draining LN (dLN) while also activating innate immune responses at the injection site. After uptake, ZIF-8 NPs undergo pH-responsive degradation in the endosome, allowing them to simultaneously release their payload, while the imidazole produced by their degradation activates TLRs in endosomes (TLRe). Analysis of gene expression in the dLN suggests a MyD88-dependent response and the activation of nuclear factor κB (*NF*-κ*B*), leading to the production of interleukin-6 (IL-6) and type I interferon β (IFN-β) associated with antiviral responses. This coincided with increased expression of CCR-7 and costimulatory molecules such as CD80 on TLR-responsive macrophage and dendritic cell (DC) populations. When administered to mice while encapsulating RBD, ZIF-8 allows for dose sparing compared to free RBD and the ability to coencapsulate with a TLR-7 agonist adjuvant, gardiquimod (Gdq). ZIF-8 also improves the stability of RBD in storage.

## RESULTS

### ZIF-8 encapsulates Gdq and RBD trimer and exhibits pH-responsive release

**Fig. 1.**
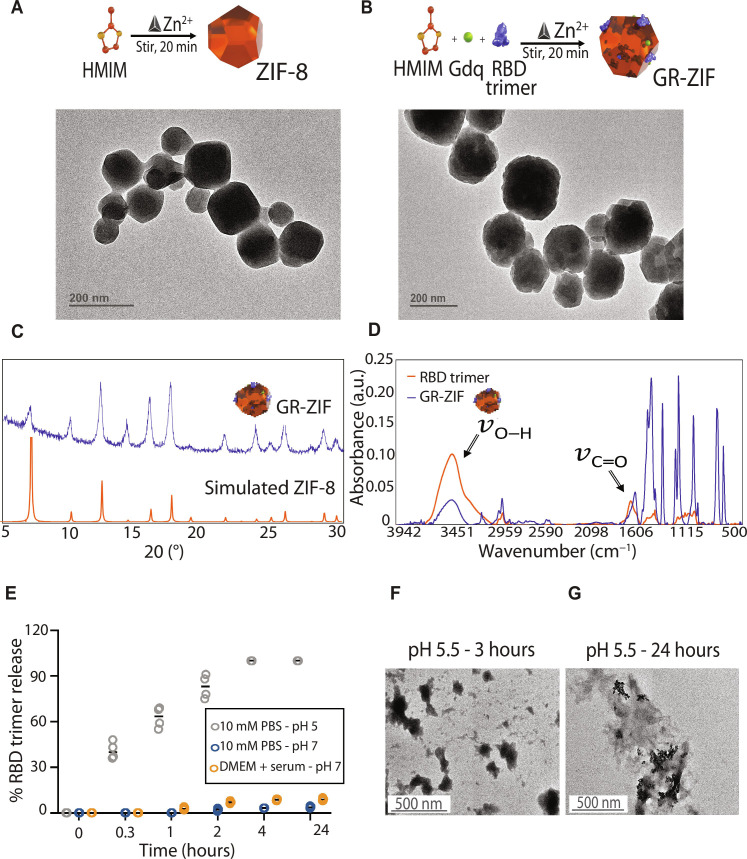
ZIF-8 encapsulate RBD trimer and Gdq. (**A** and **B**) TEM micrographs and simplified schematic of ZIF-8 (A) and GR-ZIF (B). (**C**) PXRD patterns of simulated ZIF-8 and GR-ZIF, demonstrating the retained crystallinity of ZIF-8 after loading RBD trimer and Gdq. (**D**) FTIR analyses of RBD trimer and GR-ZIF, indicating the adsorption of RBD trimer on the surface of ZIF-8. a.u., arbitrary units. (**E**) Cumulative release profile of RBD trimer from GR-ZIF at physiological pH (7) and endosomal pH (5.5), demonstrating the pH responsive release from ZIF-8. (**F** and **G**) TEM micrographs of GR-ZIF degradation over time in response to low pH (5.3).

ZIF-8 was prepared as described previously (*22*). Transmission electron microscopy (TEM) revealed cubic crystals of ZIF-8 encapsulating Gdq and RBD trimer (GR-ZIF) with an average size of 150 nm that is similar to that of ZIF-8 (Fig. 1, A and B). Likewise, the powder x-ray diffraction (PXRD) pattern of GR-ZIF showed a similar pattern to that of simulated ZIF-8 (Fig. 1C). The Fourier transform infrared (FTIR) spectrum of GR-ZIF spectrum depicted obvious stretching vibrations of C═O at 1606 cm^−1^ and O─H at 3451 cm^−1^, which were attributed to RBD trimer (Fig. 1D). FTIR analysis demonstrated that RBD trimer is not only encapsulated within ZIF-8 but also adsorbed on the surface (Fig. 1D), which allows for the enhanced presentation of RBD trimer. The uniformity and intercalation of Gdq and RBD trimer in ZIF-8 were also validated (text S1 and fig. S1).

The acidic environment of lysosomes and endosomes in APCs is expected to induce the release of RBD trimer and Gdq from ZIF-8 (Fig. 1, E and F). The pH-responsive RBD trimer release from ZIF-8 was monitored by enzyme-linked immunosorbent assay (ELISA) at physiological (pH 7.4) and lyso/endosomal acidic (pH 5.3) conditions. Under physiological conditions, less than 3% of RBD trimer was released over 24 hours in solution (Fig. 1E). Screening R-ZIF in a proteinaceous environment [Dulbecco's Modified Eagle Medium (DMEM) supplemented with 10% fetal bovine serum (pH 7.2)] indicated that 6% of RBD trimer was released from ZIF-8 in 4 hours with no further release observed after 24 hours. However, a total of 43 and 60% of RBD trimer were released at acidic pH after 20 and 120 min, respectively (Fig. 1E). TEM images confirmed that low pH triggers the degradation process for GR-ZIF. Its complete decomposition after 24 hours (Fig. 1, F and G) agrees with previously reported data (*22*).

### ZIF-8 potentiates innate immunity through the biomimetic presentation of RBD trimer

**Fig. 2.**
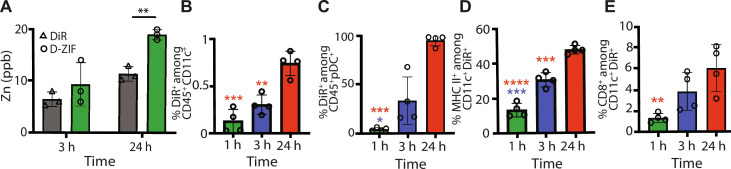
RBD trimer presentation is promoted through the rapid GR-ZIF drainage to dLN by a cell-independent and -dependent mechanisms. (**A**) C57BL/6 mice were intradermally injected with either soluble dye (XenoLight DiR) or D-ZIF. ICP-MS analysis of Zn^2+^ in dLN isolated 3 and 24 hours after intradermal injection with soluble dye or D-ZIF showing the increase in Zn^2+^ in dLN in D-ZIF–treated mice. ppb, parts per billion. (**B** to **E**) Flow cytometry quantification of CD45^+^ CD11c^+^ (B), pDCs (C), cDC^+^ MHC II^+^ (D), and cDC^+^ CD8^+^ (E) cells in dLNs 1, 3, and 24 hours after injection showing the time-dependent increase in the influx of different subsets of DCs. Data are shown as means ± SEM. *n* = 3 or 4. Statistical significance was calculated by unpaired two-tailed Student’s *t* test or one-way analysis of variance (ANOVA): *P < 0.05, **P < 0.01, ***P < 0.001, and ****P < 0.0001.

We first assessed ZIF-8 accumulation in dLN and subsequent immune activation using a series of experiments to characterize ZIF-8 biodistribution. A model cargo, XenoLight DiR, was loaded in ZIF-8 (D-ZIF) to assess the temporal localization of ZIF-8 in the dLN. Measuring Zn^2+^ in dLNs using inductively coupled plasma mass spectrometry (ICP-MS) confirmed a significant increase (*P* < 0.01) in Zn^2+^ 24 hours after injection (Fig. 2A). Flow cytometry analysis of APCs in dLN demonstrates that levels of CD45^+^ CD11c^+^ associated with D-ZIF continuously increased over time (Fig. 2B). The accumulation of D-ZIF in CD45^+^ CD11c^+^ DCs within 1 hour indicates intercellular drainage, while the significant increase in the proportion of CD45^+^ CD11c^+^ DCs associated with D-ZIF signal at 24 hours indicates intracellular drainage. This trafficking was also observed in pathogens (*28*) and other adjuvants such as MF59 adjuvant (*29*) and in agreement with other reports for submicron particles (*14*, *30*).

The activation of innate immune cells is essential for potent vaccine responses. We, therefore, investigated the capacity of ZIF-8 to activate DCs professional APCs that recognize viral antigens (*31*, *32*). Levels of CD11c^+^ CD8^+^ DC and CD11c^+^ MHC II^+^ DC increased significantly 3 and 24 hours after immunization in dLNs (Fig. 2, D and E), corroborating in vitro studies with DCs treated with ZIF-8 (text S2 and figs. S3 and S4). Plasmacytoid dendritic cells (pDCs) in particular participate in the first line of defense against viral infections by acting as innate effector cells, which initiate type-I IFN antiviral responses (*33*). They cooperate with Conventional type 1 dendritic cells (cDC.1) in LN to induce antiviral CD8^+^ T cells (*33*). Temporal trafficking of pDC in dLN revealed a significant increase in pDC levels associated with D-ZIF at 3 and 24 hours (Fig. 2C and fig. S5A). While some studies have demonstrated the ability of ZIF-8 to promote antigen and adjuvant delivery (*23*), the underlining immunological mechanisms are not fully understood.

### pH-responsive degradation of ZIF-8 in endosomes releases imidazole subunits that directly engage with the activation of TLRe and induce proinflammatory responses

Imidazole, the subunit building block of ZIF-8, is a moiety found in many commercially available TLR agonists, such as resiquimod and Gdq (*34*, *35*). We hypothesized that APCs may be activated by ZIF-8 degradation into imidazole subunits after endocytosis, the dominant uptake mechanism by which APCs take up ZIF-8. The acidic environment of endosomes induces ZIF-8 degradation, resulting in the release of its imidazole subunits and payload. Given the role of DCs in activating immune response (*36*, *37*), ZIF-8 internalization and endosomal escape were first assessed in DCs in vitro (text S2 and fig. S3). To analyze the impact of releasing ZIF-8 subunits in endosomes upon its dissociation at acidic environment, we investigated the activation status of TLR-7, TLR-8, and TLR-9 using an in vitro quantitative TLR reporter system. While Gdq is known to selectively activate TLR-7 at any concentration and TLR-8 only at high concentration, ZIF-8 activated TLR-7 at any given concentration and TLR-8 and TLR-9 only at high concentration (text S3 and fig. S6).

### ZIF-8 and GR-ZIF activate TLR expression in dLNs

**Fig. 3.**
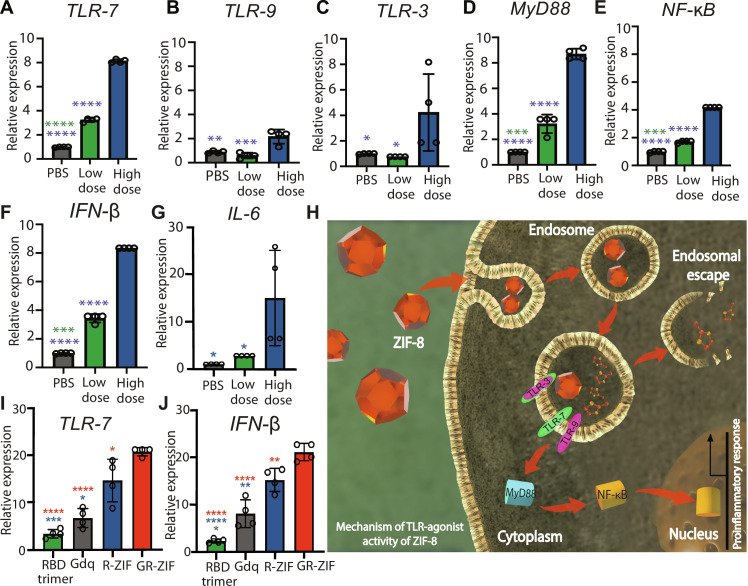
ZIF-8 induces TLR-7, TLR-9, and TLR-3 expression and initiates proinflammatory responses. C57BL/6 mice were immunized intradermally with high (200 μg) and low (25 μg) doses of ZIF-8. dLNs were harvested 24 hours after immunization for RT-qPCR. (**A** to **G**) RT-qPCR analysis of *TLR-7* (A), *TLR-9* (B), *TLR-3* (C), *MyD88* (D), *NF*-κ*B* (E) *IFN*-β (F), and *IL-6 *(G) indicating that ZIF-8 activates TLR-7 at any given concentration and TLR-3 and TLR-9 only at high concentration. (**H**) Schematic illustration of TLR agonist activity of ZIF-8 that starts by activating TLRe (TLR-7, TLR-9, and TLR-3), resulting in activating NF-κB and enhancing proinflammatory responses through MyD88-dependent pathway. (**I** and **J**) C57BL/6 mice were immunized intradermally with 3 μg of RBD trimer in solution, 0.8 μg of Gdq or 0.9 μg of RBD trimer displayed on ZIF-8 with or without Gdq. dLNs were harvested 24 hours after immunization for RT-qPCR analysis of TLR-7 (I) and IFN-β (J) expression. Data are shown as means ± SEM. *n* = 3 or 4. Statistical significance was calculated by unpaired two-tailed Student’s *t* test or one-way ANOVA: *P < 0.05, **P < 0.01, ***P < 0.001, and ****P < 0.0001.

We next assessed the expression of *TLR-3*, *TLR-7*, and *TLR-9* in dLNs harvested 24 hours after injection from mice immunized with phosphate-buffered saline (PBS) or ZIF-8 alone using reverse transcription quantitative polymerase chain reaction (RT-qPCR). *TLR-7* expression increased by four and eight times in dLN of mice immunized with low (25 μg) and high (200 μg) doses of ZIF-8, respectively (Fig. 3A). *TLR-3* and *TLR-9* expression increased (*P* < 0.05) only at a high dose of ZIF-8, indicating the importance of ZIF-8 accumulation in endosomes for the cross-activation of other TLRe (Fig. 3, B and C). To validate our results, we evaluated the expression of several target genes involved in the TLR-dependent signaling pathway such as *MyD88* and *NF*-κ*B*. Both doses of ZIF-8 significantly (*P* < 0.001) increased *MyD88* and *NF-*κ*B* expression (*P* < 0.0001; Fig. 3, D and E).

Further, in studies using vaccine antigens and adjuvants, incorporating Gdq and RBD in ZIF-8 induced greater (*P* < 0.001) *TLR-7* expression (Fig. 3I) and *TLR-9* expression (fig. S7A) than RBD or Gdq alone. The activation of TLR-9 in response to R-ZIF and GR-ZIF may suggest synergy associated with combining ZIF-8 and Gdq. The expression of *MyD88* and *NF-*κ*B* was also higher in GR-ZIF–treated mice compared to a high dose of ZIF-8 (fig. S7, B and C). RBD trimer induced slight increase in the expression of *TLR-7* but failed to activate *TLR-9*, *MyD88*, and *NF-*κ*B* (Fig. 3I and fig. S7, A to C). The activation of multiple TLRe has the potential to drive more robust cellular and humoral responses (*38*).

MyD88 is an essential adaptor in most TLRs (*7*). Therefore, we investigated the specificity of TLRe activation in response to ZIF-8 by quantifying the expression of *TLR-4*. TLR-4 recognizes polysaccharides and should not be susceptible to cross-activation at high concentrations of ZIF-8 such as TLR-3, TLR-7, TLR-8, and TLR-9, which all recognize nucleic acids—further supporting the specificity of the ZIF-8 activation mechanism. The expression of *TLR-4* was not affected by ZIF-8 or GR-ZIF (*P* > 0.05; fig. S7D), confirming the selectivity of ZIF-8 TLR activity. We also assessed gene expression of several cytokines indicative of specific TLR signaling cascades: *IFN-*β–induced and *IL-6*. ZIF-8, R-ZIF, and GR-ZIF significantly increased *IFN-*β (*P* < 0.0001) and *IL-6* (*P* < 0.05) expression compared to PBS control, Gdq, or RBD trimer (Fig. 3, F, G, and J, and fig. S7E).

**Fig. 4.**
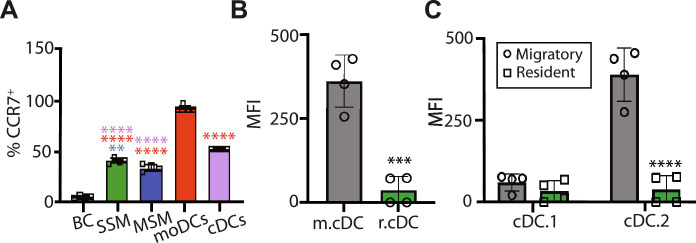
ZIF-8 activates TLR-responsive APCs. (**A**) Quantitative analysis of the proportion of CCR7^+^ APCs (migratory) to CCR7^−^ APCs (resident) in dLNs. (**B** and **C**) Quantitative analysis of the proportion of different migratory and resident DCs subsets. MFI, mean fluorescence intensity. Data are shown as means ± SEM. *n* = 4. Statistical significance was calculated by unpaired two-tailed Student’s *t* test or one-way ANOVA: **P < 0.01, ***P < 0.001, and ****P < 0.0001.

NF-κB has been identified as a key regulator of TLR-induced DC maturation. It leads to the up-regulation of CCR7, MHC II, and costimulatory molecules such as CD80 (*39*). We thus investigated the activation status of leukocytes in response to ZIF-8 using D-ZIF. CCR7 is the chemokine receptor required for the migration of leukocytes from peripheral tissue to LNs. A significant fraction of monocyte-derived DCs (moDCs), cDCs, subcapsular sinus macrophage (SSM), and marginal sinus macrophage (MSM) associated with D-ZIF signal in dLN was migratory (CCR7^+^) (Fig. 4A and fig. S5A). Further stratification of cDC subsets revealed that migratory DCs (m.DCs) accounted for the majority of cDCs in dLN (Fig. 4B). Migratory cDC.2 was significantly increased (*P* < 0.0001), suggesting that the majority of cDC.2 in dLN was of myeloid origin (Fig. 4C). Compared to PBS controls, D-ZIF induced the up-regulation of CD80 (B7-2) in B cells, SSMs, MSMs, moDCs, and migratory conventional DCs (m.cDC) (fig. S5B). A significant (*P* < 0.05) increase in CD80 expression was also observed in m.cDC.2 (fig. S5C). No significant expression in CD80 was associated with cDC.1 as CD8^+^ DCs do not respond to TLR7-mediated stimuli (*40*), explaining the markedly high expression of CD80 in leukocytes associated with high levels of CCR7.

Methylimidazole (HMIM) is the smallest moiety in synthetic TLR agonists and nitrogenous bases comprise both RNA and DNA (*26*, *27*). We therefore used an in vitro quantitative TLR reporter system and in vivo RT-qPCR to probe the connection between HMIM and TLR agonist activity of ZIF-8. HMIM showed a similar activity of ZIF-8 in which it activated TLR-7 reporter cells after 24 hours at any given concentration and TLR-8 and TLR-9 reporter cells only at high concentration (fig. S6G). Zn^2+^ showed no activation in TLR-7, TLR-8, or TLR-9 reporter cells (fig. S6H), indicating that ZIF-8 immunogenicity is mainly driven by HMIM degradation products. To assess the activation of TLR-7 in vivo, dLNs of mice injected with HMIM were harvested after 24 hours for RT-qPCR analysis. TLR-7 was up-regulated (*P* < 0.05) in response to HMIM (fig. S7F). HMIM is a small and hydrophilic molecule that is expected to diffuse rapidly to the systemic circulation. This likely contributed to the low TLR-7 expression compared to ZIF-8. Thus, ZIF-8 immunomodulatory properties are, in part, influenced by HMIM, indicating that the chemical building blocks of ZIF-8 play a major role in augmenting the immunogenicity of the carrying antigen (Fig. 3H).

To compare ZIF-8 to another vaccine delivery vehicle, we studied the possible activation of TLRe in response to mRNA-LNP. mRNA-LNP had no significant influence on the expression of *TLR-7* (fig. S8C) at the used dose but significantly (*P* < 0.01) enhanced *TLR-9* and *NF-*κ*B* expression (fig. S8, D and E). Note that mRNA loaded in LNP codes for the full spike protein, which is known to contribute to the self-adjuvant properties of mRNA vaccines. Unlike ZIF-8, mRNA-LNP immunogenicity is mainly driven by moDCs and m.cDC.1 (text S4 and fig. S8). This suggests that the TLR-7–responsive properties of ZIF-8 may be somewhat unique among potential nanoscale vaccine delivery vehicles.

To assess the translatability of GR-ZIF, we examined its safety and stability. GR-ZIF did not induce reactive oxygen species production nor apoptosis at the given concentration in mice, and the body well accommodated the increase in Zn^2+^ (text S5 and fig. S9). ZIF-8 improved RBD stability in both dry and solution states at room temperature and 4°C over 2 months (fig. S10 and text S6). The facile synthesis of GR-ZIF and improved stability of RBD trimer could potentially enable easier technology transfer to low-income countries and other areas with limited infrastructure.

### ZIF-8 encapsulating RBD trimer promotes humoral and cellular immune responses

**Fig. 5.**
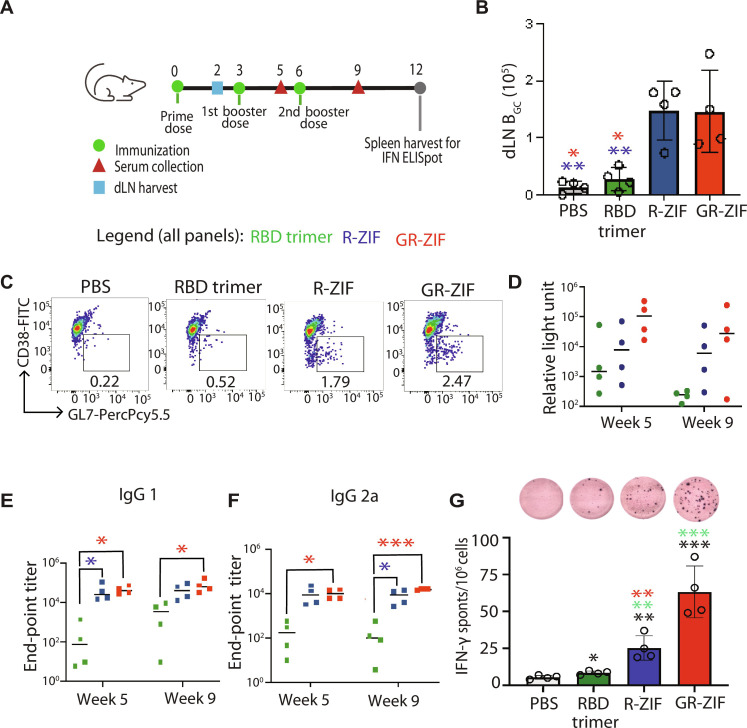
ZIF-8 induces potent cellular and humoral immune responses at a modest dose of 0.9 μg of RBD trimer in vivo. (**A**) The timeline of vaccine immunization and serum collection. C57BL/6 mice were immunized intradermally with 3 μg of soluble RBD trimer or 0.9 μg of R-ZIF or GR-ZIF and boosted on weeks 3 and 6 with the same formulations. The levels of RBD-specific IgG in the sera collected on weeks 2, 5, and 9 after immunization were detected with ELISA. dLNs were harvested for B_GC_ assessment by flow cytometry on week 2 following prime dose. Spleens were harvested on week 12 after immunization to assess memory responses by IFN-γ^+^ ELISpot. (**B** and **C**) Quantification (B) and representative flow plots (C) of B_GC_ cells (B220^+^, GL7^hi^, and CD38^lo^) in dLN. FITC, fluorescein isothiocyanate. (**D**) WA1/2020 RBD-specific IgG calculated after logarithmic transformation (log_10_) of antibody levels. (**E** and **F**) RBD-specific IgG1 (E) and IgG2 (F) as quantified by ELISA. (**G**) ELISpot analysis of IFN-γ spot-forming cells in splenocytes after ex vivo stimulation to assess memory responses. Data are shown as means ± SEM. *n* = 3 or 4. Statistical significance was calculated by unpaired two-tailed Student’s *t* test or one-way ANOVA: *P < 0.05, **P < 0.01, and ***P < 0.001.

We hypothesized that the immunostimulatory properties of ZIF-8 NPs would make them a useful platform for subunit vaccines. First, we assessed the capacity of RBD trimer, R-ZIF, and GR-ZIF to stimulate humoral responses. Brachial dLNs were harvested 2 weeks following injection to analyze germinal center B cell (B_GC_) responses. R-ZIF and GR-ZIF elicited B_GC_ responses greater than RBD trimer (Fig. 5, B and C, and fig. S11), suggesting the substantial impact of ZIF-8 in potentiating subunit protein immunogenicity.

We then vaccinated C57BL/6 mice with three injections of either RBD trimer (3 μg), R-ZIF, or GR-ZIF (containing 0.9 μg of RBD trimer) at week 0, 3, and 6 (Fig. 5A). To investigate RBD-specific immunoglobulin G (IgG), serum was collected 2 weeks after the first booster doses and 3 weeks after the second booster dose as illustrated in Fig. 5A. Median RBD-specific total IgG responses following both booster doses were high in R-ZIF and GR-ZIF but low in RBD trimer (Fig. 5D). Unlike RBD trimer, R-ZIF and GR-ZIF elicited balanced, high-titer, IgG1, and IgG2a serum responses (Fig. 5, E and F). While the incorporation of Gdq induced TLR-7 and IFN-β expression as indicated in Fig. 3 (I and J), R-ZIF and GR-ZIF exhibited comparable B_GC_ responses and yielded comparable IgG1 and IgG2a titers after the first booster dose (Fig. 5, B to F). Together, these results indicate that the use of ZIF-8 in a vaccine formulation enhances antigen immunogenicity.

SARS-CoV-2–specific cellular responses were assessed by enzyme-linked immunospot (ELISpot). Three weeks following the second booster dose, splenocytes were restimulated ex vivo with RBD trimer to evaluate IFN-γ–producing cells. Mice treated with GR-ZIF showed eightfold increase in the number of IFN-γ counts on average compared with PBS controls (*P* < 0.0001; Fig. 5F). In contrast, animals immunized with RBD trimer failed to induce IFN-γ release and, by extension, antigen-specific T cell responses (Fig. 5G), suggesting the impact of using ZIF-8 in improving memory responses.

### Transcriptome analysis of dLNs in vaccinated mice reveals that GR-ZIF enhanced the activation of innate signaling pathways

To understand differences between transcriptional responses of RBD trimer and GR-ZIF and to isolate the role of ZIF-8 in tuning immune responses, RNA sequencing (RNA-seq) was performed. dLNs were collected at the end of the first week after the first booster dose for analysis. Since GR-ZIF showed rapid induction of adaptive immune responses and R-ZIF yielded B_GC_ responses and titers similar to GR-ZIF after the first booster dose, we sought to compare transcriptional responses of GR-ZIF to RBD trimer. Principal components analysis of the transcriptomes for all vaccines revealed grouping of responses away from the PBS control (fig. S12A). Further RNA-seq global transcriptome analysis identified immune-related transcriptomic pathways affected by the immunization. The gene set enrichment analysis (GSEA) (*41*) showed significant up-regulation of 17 pathways in GR-ZIF–immunized mice. These pathways were related to antigen presentation on APC, T cell activation, and IFN-γ production (fig. S12B).

**Fig. 6.**
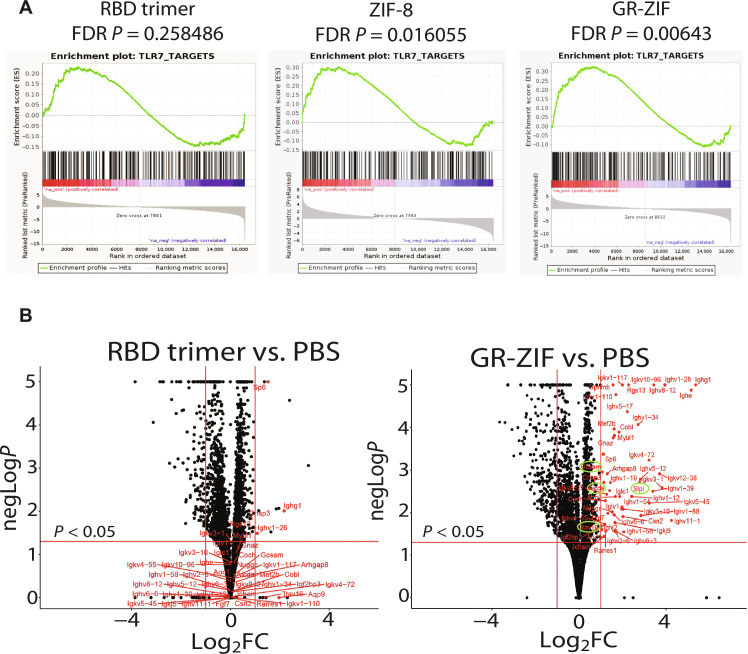
RNA-seq analyses of dLNs of C57BL/6 mice immunized intradermally with 3 μg of RBD trimer in solution, ZIF-8, or 0.9 μg of RBD trimer displayed on ZIF-8 with Gdq. One week following first booster dose, dLNs were harvested for RNA-seq. (**A**) GSEA plots showing enrichment of TLR-7–related gene sets in RBD trimer, ZIF-8, and GR-ZIF showing the up regulation of processes related to TLR7 in ZIF-8– and GR-ZIF–treated mice. (**B**) Volcano plot showing variance in gene expression with respect to fold change (FC) and significance (*P* < 0.05) in dLN of RBD trimer– and GR-ZIF–treated mice. Each dot represents an individual gene: Red dots represent the up-regulated genes in GR-ZIF.

Unexpectedly, our differential GSEA revealed that TLR-7 was still enriched after a week of immunizing mice with ZIF-8 [false discovery rate (FDR) < 0.05; Fig. 6A and fig. S12A]. Loading RBD trimer and Gdq in ZIF-8 induced better enrichment of TLR-7 (FDR < 0.05; Fig. 6A) but not in soluble RBD trimer (FDR > 0.05; Fig. 6A). To validate data obtained from the RNA-seq analysis, we performed RT-qPCR on selected genes *TLR-7* and *TLR-9*. The expression of *TLR-7* and *TLR-9* for R-ZIF– and GR-ZIF–treated mice significantly increased (fig. S12, B and C), while no significant increase was observed in RBD trimer–immunized mice. Together, these data suggest an intrinsic adjuvant effect of the ZIF-8 carrier in activating innate signaling pathways, which can be further enhanced with codelivery of a TLR agonist.

We next assessed the differentially expressed genes in the dLNs of mice given GR-ZIF or RBD trimer and compared to those injected with PBS. Volcano plot analysis identified significant up-regulation of 55 genes related to innate, B and T cell genes in GR-ZIF–immunized mice (Fig. 6B). Most of the genes were specifically up-regulated in GR-ZIF versus PBS but not RBD trimer versus PBS. Some of the most highly differentially expressed genes included activation-induced cytidine deaminase (*AICDA*) (mediates antibody class switching and somatic hypermutation in B cells) (*42*), germinal center associated signaling and motility (*GCSAM*, regulator of B cell receptor signaling) (*43*), secretory leukocyte peptidase inhibitor (*SLPI*, plays a role in regulating the activation of NF-κB and inflammatory responses) (*44*), and T cell receptor alpha variable 16 (*TRAV16*, αβ T cell receptors recognize peptide-MHCs displayed by APC) (*45*, *46*). To confirm whether R-ZIF induces a similar expression profile as GR-ZIF, *AICDA* and *TRAV16* expression was analyzed using RT-qPCR. The RT-qPCR analysis validated the expression of *AICDA* and *TRAV16 *in the dLNs of R-ZIF–treated mice (fig. S14). Together, these data suggest an intrinsic adjuvant effect of the ZIF-8 carrier in activating innate signaling pathways, which can be further enhanced with codelivery of a TLR agonist.

## DISCUSSION

PRR, particularly TLRs, have been shown to generate a greater response magnitude against vaccine targets (*7*, *47*). However, TLR agonists rapidly diffuse from injection site and, in some cases, can provoke fatal systemic inflammatory responses (*48*–*51*). Thus, several delivery approaches were developed to tune TLR agonist pharmacokinetics (*12*, *14*, *15*, *52*). Many studies viewed NPs as passive carriers (*53*, *54*), overlooking their potential in activating stimulatory immune pathways that could alter their immunostimulatory properties. Therefore, it is important to understand the intrinsic immune characteristics of NPs to ensure moderate activation of inflammatory responses and enable selective activation of immunologic pathways to maximize vaccine potency. Here, we investigated the influence of ZIF-8 accumulation, distribution, retention, and efficacy on immune response, which are important determinants for successful clinical translation. We demonstrated that the chemical and physical properties of ZIF-8 modulate immune responses to SARS-CoV-2 RBD trimer at the cellular and molecular levels. Simultaneously, ZIF-8 physical properties facilitated multivalent antigen display and LN drainage in a pathogen-mimetic approach. This was also observed in other U.S. Food and Drug Administration–approved NP-based vaccine platforms used to deliver SARS-CoV-2 spike protein, such as micelle-attached spike (NVX-CoV 2373) (*5*).

We also demonstrated that the specific chemical building blocks of ZIF-8 play a major role in augmenting the immunogenicity of the encapsulated antigen. The pH-responsive degradation of ZIF-8 into HMIM selectively engages TLR in APCs. This, in turn, activates MyD88-dependent pathways that activate NF-κB, leading to the production of IL-6 and IFN-β associated with antiviral responses and increased surface expression of CCR-7 and CD80 primarily on TLR-responsive APCs. This suggests that ZIF-8 NPs are an immunostimulatory vehicle capable of selectively activating TLRe upon cargo release. Both physical and chemical properties resulted in RBD trimer dose-sparing and efficient innate and adaptive immune responses. Future studies should address the potential impact of cellular and molecular networks activated by ZIF-8 on virus neutralizing antibody responses. Overall, our study demonstrates that probing cellular and molecular networks that drive immune responses to NPs’ composition and physical properties can reveal mechanistic insights and can potentially enhance the rational development of novel vaccines.

## MATERIALS AND METHODS

### Materials

Zinc nitrate hexahydrate (228737, purity≥98%), 2-methylimidazole (M50850, purity ≥98.5 %), and gardiquimod (SML0877, purity≥98%) were purchased from Sigma-Aldrich. SARS-CoV-2 spike RBD trimer–histidine tag was purchased from Acro Biosystems (SPN-C52H9, purity ≥95%). Purified mRNAs were obtained from TriLink BioTechnologies. d-Luciferin and Bright-Glo reagent were purchased from Promega. All other reagents were purchased from Sigma-Aldrich. 

Heptadecan-9-yl-8-{[2-hydroxyethyl][6-oxo-6-(undecyloxy)hexyl]amino}octanoate (SM-102) was purchased from Echelon, 1,2-dioleoyl-*sn*-glycero-3-phosphoethanolamine (DOPE), and 1-2-dimyristoyl-*sn*-glycero-3-phosphoethanolamine-*N*-[methoxy(polyethylene glycol)-2000] (ammonium salt) (Avanti, C14-PEG2000) were purchased from Avanti Polar Lipids. LysoTracker, Hoechst 33342, XenoLight DiR, and ProLong diamond antifade mountant were purchased from Thermo Fisher Scientific. Spleen dissociation medium was obtained from STEMCELL Technologies.

### GR-ZIF preparation and characterization

GR-ZIF was synthesized by stirring Gdq (0.1 mg ml^−1^), RBD trimer (0.1 mg ml^−1^), and HMIM (2.5 M, 0.9 ml) for 20 min. Zinc nitrate solution (0.5 M, 0.1 ml) was slowly added under mechanical agitation for 20 min. The resulting product was collected by centrifugation and washed three times with deionized water to remove any residues. ZIF-8 was synthesized by slowly adding zinc nitrate solution (0.5 M, 0.1 ml) to HMIM (2.5 M, 0.9 ml). The solution was agitated for 20 min. The supernatant of GR-ZIF was collected to calculate the loading capacity (LC) and loading efficiency (LE) of RBD trimer by ELISA and Gdq by ultraviolet-visible. D-ZIF was synthesized by stirring DiR (0.25 mg) with HMIM, followed by the slow addition of zinc nitrate solution.

LE and LC of Gdq and RBD trimer in ZIF-8 were calculated as follows

LC=[mass loaded drug/mass of loaded drug+NPs]×100 (1)

LE=[mass of drug loaded/mass of initial drug]×100 (2)

The zeta potential of GR-ZIF was performed using a Malvern Zetasizer Nano ZS at 25°C at pH 7.3 in aqueous solutions. PXRD measurements were performed using a Panalytical X’Pert Pro X-ray powder diffractometer at the MIT Materials Research Laboratory using the Cu Kα radiation (40 V, 40 mA, λ = 1.54056 Å) in a θ-θ mode from 20° to 90° (2θ). TEM images were obtained using FEI Tecnai microscope operating at 120 kV at the MIT Materials Research Laboratory. For visualization by TEM, samples were prepared by dropping the solution on a copper grid of 300 mesh (Electron Microscopy Sciences, LC 300-Cu). To evaluate the release of RBD trimer from ZIF-8, SARS-CoV-2 spike RBD ELISA kit (Acro Biosystems) was performed. Aliquots of hydrochloric acid were added to R-ZIF (0.6 mg ml^−1^) in PBS to reach pH 5.3 at 37°C. PBS only or DMEM supplemented with 10% serum were added to the sample of pH 7.3. The supernatant of the mixture solution was obtained through centrifugation at different time points.

### mRNA-LNP synthesis

Various high-purity lipids were used for LNP synthesis, including

SM-102, DOPE, cholesterol, and C14-PEG2000. Lipids were dissolved in ethanol at a molar ratio of 50:10:38.5:1.5 SM-102:DOPE:cholestorol:C14-PEG2000 To prepare the LNPs, the ethanoic solution was rapidly added to and mixed with an mRNA solution buffered with citrate at pH 3 at volume ratio 3:1 (aqueous:ethanol).

### In vitro ZIF-8 biocompatibility

CCK-8 assay was performed according to the manufacturer’s protocol. Bone marrow–derived dendritic cells (BMDCs) were provided by the Koch Institute High Throughput Sciences Core. Briefly, BMDC (5 × 10^3^ cells per well) were seeded onto a 96-well plate. Cells were incubated with different concentrations (100, 50, 25, 12, 6, and 3 μg ml^−1^) of ZIF-8 in 200 μl of DMEM at 37°C for 24 hours. The medium was then discarded, and the prepared culture medium containing 10% CCK-8 solution was added into each well, including a negative control of culture medium alone. After 3 hours of incubation, the absorbance was measured at 450 nm using a microplate reader (Tecan Infinite 200 microplate reader).

### In vitro BMDC activation

BMDCs were seeded into a 24-well plate at 2 × 10^5^ cells per well and treated with PBS, soluble RBD trimer, or GR-ZIF at ZIF-8 (10 μg ml^−1^) for 24 hours. Cells were then collected and measured for the expression of CD80 (BioLegend, 104714-BL) and CD86 (BioLegend, 105106-BL) by flow cytometry (BD FACSCanto, USA). Cell culture supernatant was also collected after 48 hours for the measurement of DC secretion of tumor necrosis factor–α by ELISA.

### Uptake and intracellular processing of RBD trimer by DCs

For RBD trimer uptake study, DCs were seeded into a 24-well plate at 2 × 10^5^ cells per well and incubated with soluble RBD trimer or GR-ZIF at 10 μg of ZIF-8 per well for 3 or 24 hours, followed by the measurement of fluorescence intensity among CD11c^+^ DCs (BioLegend, 117328) by flow cytometry. For the confocal laser scanning microscopy (Olympus FV1200 CLSM) study, DCs were seeded on glass coverslips put into a 24-well plate and incubated with the soluble mixture of Alexa Fluor 647–bovine serum albumin (BSA) or BSA loaded ZIF-8) for 3 or 24 hours, followed by washing and staining with 0.1 μM LysoTracker and Hoechst 33342 (1 μg ml^−1^) at 37°C for 1 hour. Cells were then fixed using 4% paraformaldehyde solution, coated on a glass slide using the ProLong Diamond Antifade Mountant, and visualized using confocal laser scanning microscopy.

### Human embryonic kidney–Blue mouse TLR-7, TLR-8, and TLR9 reporter cells

Cells were purchased from InvivoGen (hkb-mtlr3, hkb-mtlr7, and hkb-mtlr9) and cultured following the manufacturer’s instructions in 96-well plate containing 5 × 10^3^ cell per well. Cells were transfected with PBS, HMIM (25 and 2.5 mM), Zn^2+^ (0.5 and 0.05 mM), RBD trimer (10, 1, and 0.1 μg), Gdq (10 and 1 μg), ZIF-8 (10 and 1 μg), and GR-ZIF (10 and 1 μg) for 24 hours. For human embryonic kidney–Blue detection, we followed the manufacturer’s instructions. The absorbance at 630 nm was assessed using a microplate reader (Tecan Infinite 2000).

### Ethics statement

All animal procedures were approved by the Institutional Animal Care and Use Committees. Six- to 8-week-old C57BL/6 female mice were purchased from Charles River Laboratories Inc. Methods were chosen to minimize pain and distress to the mice, which were observed daily by trained animal care staff. The mice were terminated using a CO_2_ inhalation chamber.

### In vivo time-course for LN drainage analysis

For GR-ZIF accumulation studies, C57BL/6 female mice were intradermally injected with XenoLight DiR (excitation/emission, 710/760 nm) or D-ZIF-8 in the right forelimb of hairless mice. For flow cytometry analysis, the brachial dLN was harvested and dissociated using a spleen dissociation medium according to the manufacturer’s instructions. Cells were centrifuged and resuspended in a buffer containing zombie violet, CD45, CD11c (Tonbo Biosciences, 910004), CD11b (Tonbo Biosciences, 910004), CD8 (Tonbo Biosciences, 910004), and MHC II (Tonbo Biosciences, 910004). Cells were then washed and resuspended in PBS + 2% BSA.

### APC staining for flow cytometry

Intradermal administration of D-ZIF was performed in the right forelimb of C57BL/6 mice using a tuberculin syringe. The brachial dLN was harvested and dissociated as described previously (*55*). The resulting single-cell suspension was stained with Live/Dead near-infrared viability stain (Thermo Fisher Scientific, Waltham, MA, USA), and fluorochrome-conjugated antibodies were adapted from Optimized Multicolor Immunofluorescence Panel 061 (OMIP 061) (*55*) as follows: Becton Dickinson (Franklin Lakes, NJ, USA): CD172a BB700 clone P84, MHCII BV711 clone M5/114, CD64 BV786 clone X54-5/7.1, CCR7 phycoerythrin (PE) clone 4B12, CD80 PE-CF594 clone 16-10A1, CD11b APC-R700 clone M1/70, Ly6G BUV737 clone 1A8, B220 BUV496 clone RA3-6B2, and CD45 BUV661 clone 30-F11; BioLegend (San Diego, CA, USA): CD11c BV421 clone N418, XCR1 BV650 clone ZET, CD3 BV563 clone 17A2, CD169 PE-Cy7 clone 3D6.112, and pCDA-1 APC clone 927; Thermo Fisher Scientific: F4/80 PE-Cy5 clone BM8. Stained cells were analyzed on a FACSymphony A3 (Becton Dickinson) at the MIT Koch Institute Swanson Biotechnology Center Flow Cytometry Facility. After gating on live single cells and lymphocytes, populations were identified according to the gating strategy and immunophenotypes listed in fig. S7 and table S1.

### In vivo pharmacokinetics and histological assays

C57BL/6 female mice were intradermally injected GR-ZIF-8 in the right forelimb of hairless mice. Blood, LN, kidneys, and spleen were all harvested and digested using aqua regia and incubated at 80°C for 2 hours. The obtained liquid was diluted and subjected to ICP-MS analysis at the MIT Materials Research Laboratory to measure Zn^2+^. In the histological assay, LNs were snap-frozen inside cryomolds using Tissue-Tek O.C.T. Compound mounting medium (Thermo Fisher Scientific, 23730571, Pittsburgh, PA).

### Immunization

C57BL/6 female mice received primary immunization and two boosts on weeks 0, 3, and 6. Mice in each group (*n* = 4) were immunized intradermally at the right forelimb with PBS, RBD trimer, R-ZIF, or GR-ZIF. Mice were bled on weeks 2, 5, and 9.

### Electrochemiluminescence assay for total IgG determination

Electrochemiluminescence assay plates [MesoScale Discovery (MSD) SARS-CoV-2 IgG catalog no. N05CA-1; panel 33] were designed and produced with one antigen spot in each well. Briefly, the antigens included WA1/2020 variant RBD protein. Plates were blocked with 50 μl of blocker A (1% BSA in distilled water) solution for 30 min at room temperature shaking at 700 rpm with a digital microplate shaker. Meanwhile, the serum was diluted 1:5000 in Diluent 100 (MesoScale Discovery). The plates were three times washed with 150 μl of wash buffer (0.5% Tween 20 in 1× PBS) and blotted dry. Then, 50 μl of the diluted samples were added in duplicate to the plates and set to shake at 700 rpm at room temperature for 2 hours. Secondary antibody was prepared using Jackson ImmunoResearch Rabbit Anti-Mouse IgG detection antibody (catalog no. 315-005-045) conjugated to the MSD GOLD SULFO-TAG by *N*-hydroxysuccinimide ester chemistry per the manufacturer’s guidelines (catalog no. R91AO-1). The plates were washed three times, and 50 μl of tagged secondary antibody solution diluted 1:1000 in Diluent 100 was added to each well and incubated shaking at 700 rpm at room temperature for at least 1 hour. Plates were washed again three times, 150 μl of MSD GOLD Read Buffer B was added to each well, and the plates were read immediately after on a MESO QuickPlex SQ 120 machine. Antibody titer for each sample was reported as relative light units (RLU) that were calculated as average sample RLU minus blank RLU for each sample. The limit of detection was defined as 1000 RLU for each assay.

### SARS-CoV-2 anti-RBD binding titers

Plates are coated with purified recombinant SARS-CoV-2 spike S1-RBD antigen (2 μg ml^−1^) in 1× PBS and incubated at 4°C overnight. After incubation, plates were washed once with a wash buffer (0.05% Tween 20 in 1× PBS) and blocked with 350 μl of BSA block per well. The block solution was discarded after 2 to 3 hours of incubation at room temperature, and plates were blotted dry. Plates were incubated with serial dilutions of sera. Goat anti-mouse IgG1 horseradish peroxidase conjugates (ab97230) or goat anti-mouse IgG2a horseradish peroxidase conjugates (ab97245) were used as secondary antibodies, and 3,5,3′,5′-tetramethylbenzidine was used as a substrate. End-point titers were calculated as the dilution that emitted an optical density exceeding 3× background produced by serum from naïve mice. Antibody titers were log-transformed before all statistical processes and are presented end-point titer.

### Intracellular cytokine staining

Single-cell spleen suspensions were harvested from immunized C57BL/6 mice by enzymatic disruption, according to the manufacturer’s protocol (STEMCELL Technologies, 07915). Red blood cell lysis buffer was used to lyse the erythrocytes (eBioscience, 00433357). Cells were centrifuged and resuspended in a buffer containing zombie violet (BioLegend, 423113), CD45 (BioLegend, 103139), CD4 (BioLegend, 100553), and CD8 (BioLegend, 344711) antibodies for 30 min at 4°C. Cells were then washed and suspended in fixation/permeabilization buffer (eBioscience, 00-5123-43) for 20 min. The single-cell suspension was then washed and incubated with anti-CD16/32 (BD Pharmingen, catalog no. 553141) to reduce nonspecific binding to the fragment crystallizable region (Fc receptor). Cells were further stained with IFN-γ (Invitrogen, 45-7311-82), washed, and resuspended in PBS + 2% BSA.

### ELISpot assay

Splenocytes from immunized animals were isolated three months following prime dose and assessed by ELISpot (R&D Systems, EL485) for IFN-γ production according to the manufacturer’s protocol. Splenocytes were added at a final concentration of 0.5 × 10^6^ per well and coincubated with recall antigens (0.1 μg per well) in complete medium for 16 hours at 37°C. Following incubation, plates were washed with the provided washing buffer, and anti-mouse IFN-γ–biotin was added overnight at 4°C. Plates were washed, and streptavidin was added for a further 2 hours at room temperature. Plates were washed with washing solution, and spots were developed using bromochloroindolyl phosphate–nitro blue tetrazolium substrate. Once plates were dry, spots were counted using ELISpot reader system.

### RNA isolation and total RNA-seq preparation

Mice were injected with RBD trimer (3 μg), ZIF-8, or GR-ZIF (containing 0.9 μg of RBD trimer) at week 0, 3, and 6. RNA was extracted from harvested brachial dLN (three biological replicates per condition) at week 5, after the first booster dose. Total RNA was isolated using RNA MiniPrep Kit (QIAGEN). Libraries were prepared using the ribosomal RNA depletion chemistry method, and whole-transcriptome sequencing was performed using NextSeq by Illumina. RNA-seq data were used to quantify transcripts from the mm10 mouse assembly with the Ensembl version 101 annotation using Salmon version 1.3.0 (*56*). Gene level summaries were prepared using tximport version 1.20.0 (*57*) running under R version 4.1 (*58*). Differential expression analysis was done with DESeq2 version 1.32.0 (*59*, *60*), and differentially expressed genes were defined as those having an absolute apeglm (*61*) log_2_ fold change greater than 1 and an adjusted *P* value less than 0.05. Data parsing and clustering were done using Tibco Spotfire Analyst 7.6.1. Mouse genes were mapped to human orthologs using Mouse Genome Informatics (www.informatics.jax.org/) orthology report, and preranked GSEA (*62*) was done using javaGSEA version 2.3.0_beta_2 with msigDb version 6.2 gene sets (*41*). RNA-seq data are available from the Gene Expression Omnibus under accession number GSE246178.

### Quantitative real-time polymerase chain reaction

Mice were injected with RBD trimer (3 μg), ZIF-8, Gdq (0.8 μg), R-ZIF or GR-ZIF (containing 0.9 μg of RBD trimer), or mRNA-LNP (containing 1 μg of mRNA) at weeks 0 and 3. RNA was extracted from harvested brachial dLN at week 5, after the first booster dose. Total RNA was isolated using RNA MiniPrep Kit (QIAGEN). cDNA was prepared with 1 μg of total using cDNA synthesis kit (QIAGEN). qPCR was performed using Roche real-time machine using cDNA template, primer mix, and SYBR green (Bio-Rad) in a final volume of 20 μl of PCR reaction. PCR primer pairs are listed in table S2.

## Supplementary Materials

### This PDF file includes:

Texts S1 to S6 Figs. S1 to S14 Tables S1 and S2 References

## References

[1] E. Caproni, E. Tritto, M. Cortese, A. Muzzi, F. Mosca, E. Monaci, B. Baudner, A. Seubert, E. De Gregorio, MF59 and Pam3CSK4 boost adaptive responses to influenza subunit vaccine through an IFN type I-independent mechanism of action. J. Immunol. 188, 3088–3098 (2012). 22351935 10.4049/jimmunol.1101764

[2] H. Lal, A. L. Cunningham, O. Godeaux, R. Chlibek, J. Diez-Domingo, S. J. Hwang, M. J. Levin, J. E. McElhaney, A. Poder, J. Puig-Barbera, T. Vesikari, D. Watanabe, L. Weckx, T. Zahaf, T. C. Heineman, Efficacy of an adjuvanted herpes zoster subunit vaccine in older adults. N. Engl. J. Med. 372, 2087–2096 (2015). 25916341 10.1056/NEJMoa1501184

[3] N. Wang, J. Shang, S. Jiang, L. Du, Subunit vaccines against emerging pathogenic human coronaviruses. Front. Microbiol. 11, 298 (2020). 32265848 10.3389/fmicb.2020.00298 PMC7105881

[4] A. Bershteyn, M. C. Hanson, M. P. Crespo, J. J. Moon, A. V. Li, H. Suh, D. J. Irvine, Robust IgG responses to nanograms of antigen using a biomimetic lipid-coated particle vaccine. J. Control. Release 157, 354–365 (2012). 21820024 10.1016/j.jconrel.2011.07.029 PMC3811132

[5] C. Keech, G. Albert, I. Cho, A. Robertson, P. Reed, S. Neal, J. S. Plested, M. Zhu, S. Cloney-Clark, H. Zhou, G. Smith, N. Patel, M. B. Frieman, R. E. Haupt, J. Logue, M. McGrath, S. Weston, P. A. Piedra, C. Desai, K. Callahan, M. Lewis, P. Price-Abbott, N. Formica, V. Shinde, L. Fries, J. D. Lickliter, P. Griffin, B. Wilkinson, G. M. Glenn, Phase 1-2 trial of a SARS-CoV-2 recombinant spike protein nanoparticle vaccine. N. Engl. J. Med. 383, 2320–2332 (2020). 32877576 10.1056/NEJMoa2026920 PMC7494251

[6] S. G. Reed, M. T. Orr, C. B. Fox, Key roles of adjuvants in modern vaccines. Nat. Med. 19, 1597–1608 (2013). 24309663 10.1038/nm.3409

[7] B. Pulendran, P. S. Arunachalam, D. T. O'Hagan, Emerging concepts in the science of vaccine adjuvants. Nat. Rev. Drug Discov. 20, 454–475 (2021). 33824489 10.1038/s41573-021-00163-y PMC8023785

[8] R. Medzhitov, Toll-like receptors and innate immunity. Nat. Rev. Immunol. 1, 135–145 (2001). 11905821 10.1038/35100529

[9] G. P. Amarante-Mendes, S. Adjemian, L. M. Branco, L. C. Zanetti, R. Weinlich, K. R. Bortoluci, Pattern recognition receptors and the host cell death molecular machinery. Front. Immunol. 9, 2379 (2018). 30459758 10.3389/fimmu.2018.02379 PMC6232773

[10] S. Akira, S. Uematsu, O. Takeuchi, Pathogen recognition and innate immunity. Cell 124, 783–801 (2006). 16497588 10.1016/j.cell.2006.02.015

[11] S. P. Kasturi, I. Skountzou, R. A. Albrecht, D. Koutsonanos, T. Hua, H. I. Nakaya, R. Ravindran, S. Stewart, M. Alam, M. Kwissa, F. Villinger, N. Murthy, J. Steel, J. Jacob, R. J. Hogan, A. Garcia-Sastre, R. Compans, B. Pulendran, Programming the magnitude and persistence of antibody responses with innate immunity. Nature 470, 543–547 (2011). 21350488 10.1038/nature09737 PMC3057367

[12] G. M. Lynn, C. Sedlik, F. Baharom, Y. Zhu, R. A. Ramirez-Valdez, V. L. Coble, K. Tobin, S. R. Nichols, Y. Itzkowitz, N. Zaidi, J. M. Gammon, N. J. Blobel, J. Denizeau, P. de la Rochere, B. J. Francica, B. Decker, M. Maciejewski, J. Cheung, H. Yamane, M. G. Smelkinson, J. R. Francica, R. Laga, J. D. Bernstock, L. W. Seymour, C. G. Drake, C. M. Jewell, O. Lantz, E. Piaggio, A. S. Ishizuka, R. A. Seder, Peptide-TLR-7/8a conjugate vaccines chemically programmed for nanoparticle self-assembly enhance CD8 T-cell immunity to tumor antigens. Nat. Biotechnol. 38, 320–332 (2020). 31932728 10.1038/s41587-019-0390-x PMC7065950

[13] B. Sun, T. Xia, Nanomaterial-based vaccine adjuvants. J. Mater. Chem. B 4, 5496–5509 (2016). 30774955 10.1039/C6TB01131D PMC6377210

[14] G. M. Lynn, R. Laga, P. A. Darrah, A. S. Ishizuka, A. J. Balaci, A. E. Dulcey, M. Pechar, R. Pola, M. Y. Gerner, A. Yamamoto, C. R. Buechler, K. M. Quinn, M. G. Smelkinson, O. Vanek, R. Cawood, T. Hills, O. Vasalatiy, K. Kastenmuller, J. R. Francica, L. Stutts, J. K. Tom, K. A. Ryu, A. P. Esser-Kahn, T. Etrych, K. D. Fisher, L. W. Seymour, R. A. Seder, In vivo characterization of the physicochemical properties of polymer-linked TLR agonists that enhance vaccine immunogenicity. Nat. Biotechnol. 33, 1201–1210 (2015). 26501954 10.1038/nbt.3371 PMC5842712

[15] M. Silva, Y. Kato, M. B. Melo, I. Phung, B. L. Freeman, Z. Li, K. Roh, J. W. Van Wijnbergen, H. Watkins, C. A. Enemuo, B. L. Hartwell, J. Y. H. Chang, S. Xiao, K. A. Rodrigues, K. M. Cirelli, N. Li, S. Haupt, A. Aung, B. Cossette, W. Abraham, S. Kataria, R. Bastidas, J. Bhiman, C. Linde, N. I. Bloom, B. Groschel, E. Georgeson, N. Phelps, A. Thomas, J. Bals, D. G. Carnathan, D. Lingwood, D. R. Burton, G. Alter, T. P. Padera, A. M. Belcher, W. R. Schief, G. Silvestri, R. M. Ruprecht, S. Crotty, D. J. Irvine, A particulate saponin/TLR agonist vaccine adjuvant alters lymph flow and modulates adaptive immunity. Sci. Immunol. 6, eabf1152 (2021). 34860581 10.1126/sciimmunol.abf1152 PMC8763571

[16] C. G. Kim, Y. C. Kye, C. H. Yun, The role of nanovaccine in cross-presentation of antigen-presenting cells for the activation of CD8^+^ T cell responses. Pharmaceutics 11, 612 (2019). 31731667 10.3390/pharmaceutics11110612 PMC6920862

[17] R. S. Oakes, L. H. Tostanoski, S. M. Kapnick, E. Froimchuk, S. K. Black, X. Zeng, C. M. Jewell, Exploiting rational assembly to map distinct roles of regulatory cues during autoimmune therapy. ACS Nano 15, 4305–4320 (2021). 33645967 10.1021/acsnano.0c07440 PMC8116774

[18] M. L. Bookstaver, Q. Zeng, R. S. Oakes, S. M. Kapnick, V. Saxena, C. Edwards, N. Venkataraman, S. K. Black, X. Zeng, E. Froimchuk, T. Gebhardt, J. S. Bromberg, C. M. Jewell, Self-assembly of immune signals to program innate immunity through rational adjuvant design. Adv. Sci. 10, e2202393 (2023). 10.1002/advs.202202393 PMC9811447 36373708

[19] M. T. Abrams, M. L. Koser, J. Seitzer, S. C. Williams, M. A. DiPietro, W. Wang, A. W. Shaw, X. Mao, V. Jadhav, J. P. Davide, P. A. Burke, A. B. Sachs, S. M. Stirdivant, L. Sepp-Lorenzino, Evaluation of efficacy, biodistribution, and inflammation for a potent siRNA nanoparticle: Effect of dexamethasone co-treatment. Mol. Ther. 18, 171–180 (2010). 19738601 10.1038/mt.2009.208 PMC2839226

[20] K. Liang, R. Ricco, C. M. Doherty, M. J. Styles, S. Bell, N. Kirby, S. Mudie, D. Haylock, A. J. Hill, C. J. Doonan, P. Falcaro, Biomimetic mineralization of metal–organic frameworks as protective coatings for biomacromolecules. Nat. Commun. 6, 7240 (2015). 26041070 10.1038/ncomms8240 PMC4468859

[21] C. Wang, G. Sudlow, Z. Wang, S. Cao, Q. Jiang, A. Neiner, J. J. Morrissey, E. D. Kharasch, S. Achilefu, S. Singamaneni, Metal-organic framework encapsulation preserves the bioactivity of protein therapeutics. Adv. Healthc. Mater. 7, e1800950 (2018). 30369102 10.1002/adhm.201800950 PMC6453541

[22] S. K. Alsaiari, S. Patil, M. Alyami, K. O. Alamoudi, F. A. Aleisa, J. S. Merzaban, M. Li, N. M. Khashab, Endosomal escape and delivery of CRISPR/Cas9 genome editing machinery enabled by nanoscale zeolitic imidazolate framework. J. Am. Chem. Soc. 140, 143–146 (2018). 29272114 10.1021/jacs.7b11754

[23] Y. W. Zhang, F. Wang, E. Ju, Z. Liu, Z. Chen, J. Ren, X. Qu, Metal-organic-framework-based vaccine platforms for enhanced systemic immune and memory response. Adv. Funct. Mater. 26, 6454–6461 (2016).

[24] G. Zhang, X. Fu, H. Sun, P. Zhang, S. Zhai, J. Hao, J. Cui, J. M. Hu, Poly(ethylene glycol)-mediated assembly of vaccine particles to improve stability and immunogenicity. ACS Appl. Mater. Interfaces 13, 13978–13989 (2021). 33749241 10.1021/acsami.1c00706

[25] L. Wang, G. Zhang, Y. Sun, Z. Wu, C. Ren, Z. Zhang, X. Peng, Y. Zhang, Y. Zhao, C. Li, L. Gao, X. Liang, H. Sun, J. Cui, C. Ma, Enhanced delivery of TLR7/8 agonists by metal-organic frameworks for hepatitis B virus cure. ACS Appl. Mater. Interfaces 14, 46176–46187 (2022). 36206454 10.1021/acsami.2c11203

[26] Y. Yang, A. Csakai, S. Jiang, C. Smith, H. Tanji, J. Huang, T. Jones, K. Sakaniwa, L. Broadwell, C. Shi, S. Soti, U. Ohto, Y. Fang, S. Shen, F. Deng, T. Shimizu, H. Yin, Tetrasubstituted imidazoles as incognito Toll-like receptor 8 a(nta)gonists. Nat. Commun. 12, 4351 (2021). 34272380 10.1038/s41467-021-24536-4 PMC8285539

[27] M. Beesu, G. Caruso, A. C. Salyer, N. M. Shukla, K. K. Khetani, L. J. Smith, L. M. Fox, H. Tanji, U. Ohto, T. Shimizu, S. A. David, Identification of a human Toll-like receptor (TLR) 8-specific agonist and a functional pan-TLR inhibitor in 2-aminoimidazoles. J. Med. Chem. 59, 3311–3330 (2016). 26966993 10.1021/acs.jmedchem.6b00023

[28] G. V. Reynoso, A. S. Weisberg, J. P. Shannon, D. T. McManus, L. Shores, J. L. Americo, R. V. Stan, J. W. Yewdell, H. D. Hickman, Lymph node conduits transport virions for rapid T cell activation. Nat. Immunol. 20, 602–612 (2019). 30886418 10.1038/s41590-019-0342-0 PMC6474694

[29] S. Calabro, M. Tortoli, B. C. Baudner, A. Pacitto, M. Cortese, D. T. O'Hagan, E. De Gregorio, A. Seubert, A. Wack, Vaccine adjuvants alum and MF59 induce rapid recruitment of neutrophils and monocytes that participate in antigen transport to draining lymph nodes. Vaccine 29, 1812–1823 (2011). 21215831 10.1016/j.vaccine.2010.12.090

[30] V. Manolova, A. Flace, M. Bauer, K. Schwarz, P. Saudan, M. F. Bachmann, Nanoparticles target distinct dendritic cell populations according to their size. Eur. J. Immunol. 38, 1404–1413 (2008). 18389478 10.1002/eji.200737984

[31] N. Bhardwaj, A. Bender, N. Gonzalez, L. K. Bui, M. C. Garrett, R. M. Steinman, Influenza virus-infected dendritic cells stimulate strong proliferative and cytolytic responses from human CD8+ T cells. J. Clin. Invest. 94, 797–807 (1994). 8040335 10.1172/JCI117399 PMC296160

[32] A. S. McWilliam, A. M. Marsh, P. G. Holt, Inflammatory infiltration of the upper airway epithelium during Sendai virus infection: Involvement of epithelial dendritic cells. J. Virol. 71, 226–236 (1997). 8985342 10.1128/jvi.71.1.226-236.1997 PMC191043

[33] A. L. Musumeci, K. Winheim, E. Krug, What makes a pDC: Recent advances in understanding plasmacytoid DC development and heterogeneity. Front. Immunol. 10, 1222 (2019). 31191558 10.3389/fimmu.2019.01222 PMC6548821

[34] F. Ma, J. Zhang, J. Zhang, C. Zhang, The TLR7 agonists imiquimod and gardiquimod improve DC-based immunotherapy for melanoma in mice. Cell. Mol. Immunol. 7, 381–388 (2010). 20543857 10.1038/cmi.2010.30 PMC4002679

[35] S. Bhagchandani, J. A. Johnson, D. J. Irvine, Evolution of Toll-like receptor 7/8 agonist therapeutics and their delivery approaches: From antiviral formulations to vaccine adjuvants. Adv. Drug Deliv. Rev. 175, 113803 (2021). 34058283 10.1016/j.addr.2021.05.013 PMC9003539

[36] T. Worbs, S. I. Hammerschmidt, R. Forster, Dendritic cell migration in health and disease. Nat. Rev. Immunol. 17, 30–48 (2017). 27890914 10.1038/nri.2016.116

[37] A. Mildner, S. Jung, Development and function of dendritic cell subsets. Immunity 40, 642–656 (2014). 24837101 10.1016/j.immuni.2014.04.016

[38] T. H. Mogensen, Pathogen recognition and inflammatory signaling in innate immune defenses. Clin. Microbiol. Rev. 22, 240–273 (2009). 19366914 10.1128/CMR.00046-08 PMC2668232

[39] M. Rescigno, M. Martino, C. L. Sutherland, M. R. Gold, P. Ricciardi-Castagnoli, Dendritic cell survival and maturation are regulated by different signaling pathways. J. Exp. Med. 188, 2175–2180 (1998). 9841930 10.1084/jem.188.11.2175 PMC2212396

[40] C. L. Doxsee, T. R. Riter, M. J. Reiter, S. J. Gibson, J. P. Vasilakos, R. M. Kedl, The immune response modifier and Toll-like receptor 7 agonist S-27609 selectively induces IL-12 and TNF-α production in CD11c^+^CD11b^+^CD8^-^ dendritic cells. J. Immunol. 171, 1156–1163 (2003). 12874201 10.4049/jimmunol.171.3.1156

[41] A. Subramanian, P. Tamayo, V. K. Mootha, S. Mukherjee, B. L. Ebert, M. A. Gillette, A. Paulovich, S. L. Pomeroy, T. R. Golub, E. S. Lander, J. P. Mesirov, Gene set enrichment analysis: A knowledge-based approach for interpreting genome-wide expression profiles. Proc. Natl. Acad. Sci. U.S.A. 102, 15545–15550 (2005). 16199517 10.1073/pnas.0506580102 PMC1239896

[42] S. R. Park, Activation-induced cytidine deaminase in B cell immunity and cancers. Immune Netw. 12, 230–239 (2012). 23396757 10.4110/in.2012.12.6.230 PMC3566417

[43] I. Romero-Camarero, X. Jiang, Y. Natkunam, X. Lu, C. Vicente-Duenas, I. Gonzalez-Herrero, T. Flores, J. L. Garcia, G. McNamara, C. Kunder, S. Zhao, V. Segura, L. Fontan, J. A. Martinez-Climent, F. J. Garcia-Criado, J. D. Theis, A. Dogan, E. Campos-Sanchez, M. R. Green, A. A. Alizadeh, C. Cobaleda, I. Sanchez-Garcia, I. S. Lossos, Germinal centre protein HGAL promotes lymphoid hyperplasia and amyloidosis via BCR-mediated Syk activation. Nat. Commun. 4, 1338 (2013). 23299888 10.1038/ncomms2334 PMC3545406

[44] M. S. Mulligan, A. B. Lentsch, M. Huber-Lang, R. F. Guo, V. Sarma, C. D. Wright, T. R. Ulich, P. A. Ward, Anti-inflammatory effects of mutant forms of secretory leukocyte protease inhibitor. Am. J. Pathol. 156, 1033–1039 (2000). 10702419 10.1016/S0002-9440(10)64971-1 PMC1876846

[45] R. J. Brownlie, R. Zamoyska, T cell receptor signalling networks: Branched, diversified and bounded. Nat. Rev. Immunol. 13, 257–269 (2013). 23524462 10.1038/nri3403

[46] J. Rossjohn, S. Gras, J. J. Miles, S. J. Turner, D. I. Godfrey, J. McCluskey, T cell antigen receptor recognition of antigen-presenting molecules. Annu. Rev. Immunol. 33, 169–200 (2015). 25493333 10.1146/annurev-immunol-032414-112334

[47] S. Kumar, R. Sunagar, E. Gosselin, Bacterial protein Toll-like-receptor agonists: A novel perspective on vaccine adjuvants. Front. Immunol. 10, 1144 (2019). 31191528 10.3389/fimmu.2019.01144 PMC6549121

[48] M. J. McCluskie, J. L. Cartier, A. J. Patrick, D. Sajic, R. D. Weeratna, K. L. Rosenthal, H. L. Davis, Treatment of intravaginal HSV-2 infection in mice: A comparison of CpG oligodeoxynucleotides and resiquimod (R-848). Antiviral Res. 69, 77–85 (2006). 16377001 10.1016/j.antiviral.2005.10.007

[49] S. Jeong, Y. Choi, K. Kim, Engineering therapeutic strategies in cancer immunotherapy via exogenous delivery of Toll-like receptor agonists. Pharmaceutics 13, 1374 (2021). 34575449 10.3390/pharmaceutics13091374 PMC8466827

[50] N. Baxan, A. Papanikolaou, I. Salles-Crawley, R. Chowdhury, O. Dubois, J. Branca, M. G. Hasham, N. Rosenthal, S. K. Prasad, L. Zhao, S. E. Harding, S. Sattler, Characterization of acute TLR-7 agonist-induced hemorrhagic myocarditis in mice by multi-parametric quantitative cardiac MRI. Dis. Model. Mech. 12, dmm040725 (2019). 31324689 10.1242/dmm.040725 PMC6737951

[51] J. P. Vasilakos, M. A. Tomai, The use of Toll-like receptor 7/8 agonists as vaccine adjuvants. Expert Rev. Vaccines 12, 809–819 (2013). 23885825 10.1586/14760584.2013.811208

[52] Q. Yin, W. Luo, V. Mallajosyula, Y. Bo, J. Guo, J. Xie, M. Sun, R. Verma, C. Li, C. M. Constantz, L. E. Wagar, J. Li, E. Sola, N. Gupta, C. Wang, O. Kask, X. Chen, X. Yuan, N. C. Wu, J. Rao, Y. H. Chien, J. Cheng, B. Pulendran, M. M. Davis, A TLR7-nanoparticle adjuvant promotes a broad immune response against heterologous strains of influenza and SARS-CoV-2. Nat. Mater. 22, 380–390 (2023). 36717665 10.1038/s41563-022-01464-2 PMC9981462

[53] M. J. Mitchell, M. M. Billingsley, R. M. Haley, M. E. Wechsler, N. A. Peppas, R. Langer, Engineering precision nanoparticles for drug delivery. Nat. Rev. Drug Discov. 20, 101–124 (2021). 33277608 10.1038/s41573-020-0090-8 PMC7717100

[54] N. G. Lamson, A. Berger, K. C. Fein, K. A. Whitehead, Anionic nanoparticles enable the oral delivery of proteins by enhancing intestinal permeability. Nat. Biomed. Eng. 4, 84–96 (2020). 31686002 10.1038/s41551-019-0465-5 PMC7461704

[55] A. T. DiPiazza, J. P. Hill, B. S. Graham, T. J. Ruckwardt, OMIP-061: 20-color flow cytometry panel for high-dimensional characterization of murine antigen-presenting cells. Cytometry A 95, 1226–1230 (2019). 31424632 10.1002/cyto.a.23880

[56] R. Patro, G. Duggal, M. I. Love, R. A. Irizarry, C. Kingsford, Salmon provides fast and bias-aware quantification of transcript expression. Nat. Methods 14, 417–419 (2017). 28263959 10.1038/nmeth.4197 PMC5600148

[57] C. Soneson, M. I. Love, M. D. Robinson, Differential analyses for RNA-seq: Transcript level estimates improve gene-level inferences. F1000Res 4, 1521 (2015). 26925227 10.12688/f1000research.7563.1 PMC4712774

[58] R. C. Team. *R: A Language and Environment for Statistical Computing*, 2021).

[59] M. I. Love, W. Huber, S. Anders, Moderated estimation of fold change and dispersion for RNA-seq data with DESeq2. Genome Biol. 15, 550 (2014). 25516281 10.1186/s13059-014-0550-8 PMC4302049

[60] S. Anders, W. Huber, Differential expression analysis for sequence count data. Genome Biol. 11, R106 (2010). 20979621 10.1186/gb-2010-11-10-r106 PMC3218662

[61] A. Zhu, J. G. Ibrahim, M. I. Love, Heavy-tailed prior distributions for sequence count data: Removing the noise and preserving large differences. Bioinformatics 35, 2084–2092 (2019). 30395178 10.1093/bioinformatics/bty895 PMC6581436

[62] V. K. Mootha, C. M. Lindgren, K. F. Eriksson, A. Subramanian, S. Sihag, J. Lehar, P. Puigserver, E. Carlsson, M. Ridderstrale, E. Laurila, N. Houstis, M. J. Daly, N. Patterson, J. P. Mesirov, T. R. Golub, P. Tamayo, B. Spiegelman, E. S. Lander, J. N. Hirschhorn, D. Altshuler, L. C. Groop, PGC-1α-responsive genes involved in oxidative phosphorylation are coordinately downregulated in human diabetes. Nat. Genet. 34, 267–273 (2003). 12808457 10.1038/ng1180

[63] C. B. Maisonneuve, S. Philpott, D. J. De Gregorio, E., Unleashing the potential of NOD- and Toll-like agonists as vaccine adjuvants. Proc. Natl. Acad. Sci. U.S.A. 111, 12294–12299 (2014). 25136133 10.1073/pnas.1400478111 PMC4151741

[64] E. J. Hennessy, A. E. Parker, L. A. O'Neill, Targeting Toll-like receptors: Emerging therapeutics? Nat. Rev. Drug Discov. 9, 293–307 (2010). 20380038 10.1038/nrd3203

[65] P. Chen, M. He, B. Chen, B. Hu, Size- and dose-dependent cytotoxicity of ZIF-8 based on single cell analysis. Ecotoxicol. Environ. Saf. 205, 111110 (2020). 32810646 10.1016/j.ecoenv.2020.111110

[66] M. W. Hoopa, C. Riccòc, R. Mushtaqa, F. Terzopouloua, A. Chena, X.-Z. deMellob, A. Doonand, C. Falcaroc, P. Nelsona, B. Puigmartí-Luisb, J. Panéa, S., Biocompatibility characteristics of the metal organic framework ZIF-8 for therapeutical applications. Mater. Today 11, 13–21 (2018).

[67] D. J. A. Crommelin, T. J. Anchordoquy, D. B. Volkin, W. Jiskoot, E. Mastrobattista, Addressing the cold reality of mRNA vaccine stability. J. Pharm. Sci. 110, 997–1001 (2021). 33321139 10.1016/j.xphs.2020.12.006 PMC7834447

[68] N. Dumpa, K. Goel, Y. Guo, H. McFall, A. R. Pillai, A. Shukla, M. A. Repka, S. N. Murthy, Stability of vaccines. AAPS PharmSciTech 20, 42 (2019). 30610415 10.1208/s12249-018-1254-2

[69] O. S. Kumru, S. B. Joshi, D. E. Smith, C. R. Middaugh, T. Prusik, D. B. Volkin, Vaccine instability in the cold chain: Mechanisms, analysis and formulation strategies. Biologicals 42, 237–259 (2014). 24996452 10.1016/j.biologicals.2014.05.007

