# Supplementary material for: Zeolitic imidazolate frameworks activate endosomal Toll-like receptors and potentiate immunogenicity of SARS-CoV-2 spike protein trimer
Source: Sci Adv. 2024 Mar 6;10(10):eadj6380. doi: 10.1126/sciadv.adj6380 (PMC10917347; doi:10.1126/sciadv.adj6380)
Supplement: Supplementary file 1 — Texts S1 to S6 Figs. S1 to S14 Tables S1 and S2 References [file sciadv.adj6380_sm.pdf]

Science Advances
AAAS

Supplementary Materials for

Zeolitic imidazolate frameworks activate endosomal Toll-like receptors and potentiate immunogenicity of SARS-CoV-2 spike protein trimer

Shahad K. Alsaiari *et al.*

Corresponding author: Ana Jaklenec, jaklenec@mit.edu; Robert Langer, rlanger@mit.edu;
Dan H. Barouch, dbarouch@bidmc.harvard.edu

Sci. Adv. **10**, eadj6380 (2024)
DOI: 10.1126/sciadv.adj6380

This PDF file includes:

Texts S1 to S6
Figs. S1 to S14
Tables S1 and S2
References

Supplementary Text

Supplementary Text Section 1: Synthesis and characterization of GR-ZIF

GR-ZIF was prepared by first mixing RBD trimer and gardiquimod (Gdq) with 2-methylimidazole (HMIM, 2.5 M, 0.9 mL) for 10 minutes, followed by the addition of Zn nitrate (0.5 M, 0.1 mL). The solution was kept under mechanical agitation at room temperature for 20 minutes. The truncated GR-ZIF crystals were obtained by centrifugation after washing with DI water to remove all residues. The uniformity and good dispersity of GR-ZIF were validated by scanning electron microscopy (SEM, Fig. S1 A and B). ζ-potential measurements revealed a drop in the charge of ZIF-8 (from 9 ± 4 mV to 2 ± 1 mV), validating the composite formation (Fig. S1C).

The amount of adsorbed and encapsulated RBD trimer in ZIF-8 was determined by releasing RBD trimer from ZIF-8 using ethylenediaminetetraacetic acid (EDTA) that breaks the coordination bonds between Zn^{2+} and HMIM, and quantified by ELISA. The amount of RBD trimer in the GR-ZIF was found to be 4.5 ± 0.5 mg g^{-1}, which corresponds to the encapsulation of 63 ± 4 % of the initial amount of RBD trimer. A total of 4.6 ± 0.05 mg g^{-1} of Gdq was found in GR-ZIF composite as determined by UV-Vis spectroscopy (Fig. S1 D).

Supplementary Text Section 2: ZIF-8 improves RBD trimer presentation and promotes APC activation

ZIF-8 improves RBD trimer presentation. Given the role of DCs in activating immune response(*36, 37*), the quality of immunity induced by the interaction of GR-ZIF and DCs was assessed in vitro. Based on cytotoxicity testing (Fig. S2), a concentration of 10 μg mL^{-1} of GR-ZIF was used in all subsequent in vitro experiments. GR-ZIF should be internalized in DCs endosomes to reach TLR in endosomes (TLRe). AF-647-BSA was used as a model cargo to assess internalization (B-ZIF). DCs were pulsed with soluble AF-647-BSA or B-ZIF for either 3 h or 24 h followed by flow cytometry analysis, inductively coupled plasma mass spectroscopy (ICP-MS) and confocal laser scanning microscopy (CLSM). B-ZIF significantly enhanced cellular uptake at 24 h compared to soluble AF-647-BSA ($P< 0.0001$) (Fig. S3 A and B). Likewise, Zn concentration in DCs pulsed with B-ZIF increased significantly ($P< 0.05$) 24 h post transfection as determined by ICP-MS (Fig S3 C), confirming uptake.

To trigger cytokine release, antigen must be able to access the endosomal receptors of APCs(*63, 64*). Incubating B-ZIF with DCs for 24 h resulted in their accumulation in endosomes. We also observed free BSA at the 24 h time point, indicating endosomal escape (Supplementary Fig. 3D). However, incubating DCs with soluble BSA resulted in low uptake of BSA even after 24 h (Fig. S3 D). Endosomal uptake and escape are expected to promote in vivo antigen cross-presentation, which will consequently result in cytotoxic T cells activation and viral immune response induction. GR-ZIF also significantly ($P< 0.005$) enhanced CD86 (B7-1) and CD80 (B7-2) expression (Fig. S4 A-C) in DCs. Furthermore, GR-ZIF enhanced CD40 expression, which is considered a master

regulator of DCs (Fig. S4 D). After incubation, supernatants were tested by ELISA to determine the secretion of TNF-**α**. Compared to soluble RBD trimer, GR-ZIF resulted in a significant increase in the secretion of TNF-**α** (Fig. S4 E). Collectively, these results suggested that GR-ZIF increased RBD trimer and Gdq delivery to DCs and promoted antigen processing by DCs.

Supplementary Text Section 3: TLRe activation by ZIF-8

To analyze the responsiveness of TLRe to ZIF-8, we investigated the activation of TLR-7, TLR-8 and TLR-9 using an *in vitro* quantitative TLR reporter system. ZIF-8 activity was compared to Gdq that is known to selectively activate TLR-7 at any concentration, and TLR-8 only at high concentration. Unlike Gdq that activated only TLR-7 reporter cells after 24 h (Fig. S6 A and B), ZIF-8 activated TLR-7 reporter cells at any given concentration and TLR-9 reporter cells only at high concentration (Fig. S6 A and B). The high accumulation of ZIF-8 in endosomes at 24 h significantly ($P<0.01$) increased the activation of TLR-7 reporter cells (Fig S3 D). TLR-8 reporter cell activation significantly ($P<0.01$) increased as ZIF-8 accumulation increased in endosomes; however, there activation was not concentration-dependent (Fig. S6 A and B). GR-ZIF exhibited a similar activation pattern of TLR-7, TLR-8 and TLR-9 as ZIF-8 (Fig. S6 F). Unlike ZIF-8 and GR-ZIF, soluble RBD trimer slightly activated TLR-7 but failed to activate TLR-8 and TLR-9 (Fig. S6 C-E).

Supplementary Text Section 4: Adjuvant-specific properties of ZIF-8

Negatively charged LNPs of approximately 147 nm diameter; close to the size of GR-ZIF (Supplementary Fig. 8 A, B) encapsulating mRNA encoding for the full-length S glycoprotein were fabricated using ionizable lipid (SM-102), phospholipid, cholesterol and pegylated lipid. C57BL/6 were intradermally injected with mRNA-LNPs (mRNA dose 1 µg) in the upper forelimb. dLNS were harvested 24 h post-injection for flow cytometry analysis. CD80 was highly upregulated in moDCs, followed by BC and SSM (Fig. S8 F). Analysis of cDC subsets demonstrated the association of significantly high levels of mRNA-ZIF in m.cDC1 compared to all other cDC subsets (Fig. S8 G). Likewise, m.cDC1 is the only cDC subset demonstrated 9-fold increase in CD80 levels compared to PBS injected mice ($P< 0.05$, Fig. S8 H), indicating the role of m.cDC1 in mRNA-LNP delivery and subsequent immunogenicity. Together, flow cytometry analysis clearly showed that APCs activation in response to mRNA-LNP is likely mediated through moDCs and m.cDC1.

Supplementary Text Section 5: Safety and biocompatibility of GR-ZIF

Potential toxicity of ZIF-8 is mediated through reactive oxygen species (ROS) production that result from released Zn^{2+}(*65, 66*). Cell cycle, consequently, arrest in the G2/M phase due to irreversible DNA damage, resulting in initiating cellular apoptosis pathways. GSEA of ROS production (FDR > 0.05) and apoptosis (FDR > 0.05) related pathways showed insignificant enrichment in ZIF-8 and GR-ZIF immunized mice (Fig. S9 A, B). We further examined toxicity by measuring Zn accumulation in different organs using inductively coupled plasma mass

spectrometry (ICP-MS) and change in cell morphology of different tissue organs using H&E stain. Mice were intradermally injected with GR-ZIF (containing 0.9 µg of RBD trimer) and major organs (liver, spleen and kidneys) were harvested at different time points (6 h, 24 h and 48 h). Serum was also collected to measure Zn^{2+} concentration at 24 h and 48 h. ICP-MS showed that ZIF-8 accumulated only in liver 6 h post injection, however, the Zn^{2+}concentration diminished until it got back to normal levels 48 h post-injection, indicating that the body can well accommodate the increase in Zn^{2+} at the given concentration (Fig. S9 C). No increase in Zn^{2+} concentration observed in serum, spleens, or kidneys. Likewise, hematoxylin and eosin (H&E) stain of the same organs showed no discernible morphological changes in tissues of liver, spleen and kidney compared to PBS treated mice (Fig. S9 D and E). Overall, ZIF-8 stimulates TLRe and results in the activation of a signal transduction cascade via the activation of the adapter MyD88 and NF-κB. Thus, ZIF-8 is shown to have a high therapeutic potential with high margins of safety.

Supplementary Text Section 6: ZIF-8 extends RBD-trimer shelf-life at room temperature

Most vaccines today are formulated as aqueous solutions in a ready-to-use form, however, temperature is the main determinant of vaccine stability(*67, 68*). Several studies in the United States reported a total of 14-35% accidental exposures of vaccine vials labeled for storage at 2-8 °C to freezing temperatures (69). Unintended freezing of some adjuvanted vaccines in the vaccine cold chain jeopardize vaccine potency which has become a concern in the developing world(*69*). In addition, the WHO reported the loss of 2.8 million SARS-CoV-2 vaccines in five countries due to cold chain failures during the last pandemic(69). Hence, the development of thermostable vaccines is crucial for maintaining vaccine stability and reducing reliance on the cold chain.

We stored lyophilized and aqueous GR-ZIF at various temperatures. The recovery efficacy (% RBD trimer recovery) was calculated by comparing the recovered amount of RBD trimer to the RBD trimer amount prior to incubation. Storing GR-ZIF for seven days at room temperature after lyophilization resulted in the recovery of more than 80 % of RBD trimer (Fig. S10 A). More than 75 % of RBD trimer was recovered upon storing aqueous GR-ZIF for 60 days at room temperature and 4 ºC (Fig. S10 B and C). Lyophilizing GR-ZIF resulted in the recovery of 60 % of RBD trimer. Unlike GR-ZIF, storing free RBD trimer at room temperature for 60 days exhibited a recovery of 40% of the RBD trimer bioavailability (Fig. S10 C). Our results demonstrate the efficacy of ZIF-8 in improving the developed vaccine stability in both dry and solution states at room temperature and 4 ºC. The facile synthesis of GR-ZIF simplify technology transfer to low-income countries and could potentially speed up mass vaccination campaigns to reduce infection rate.

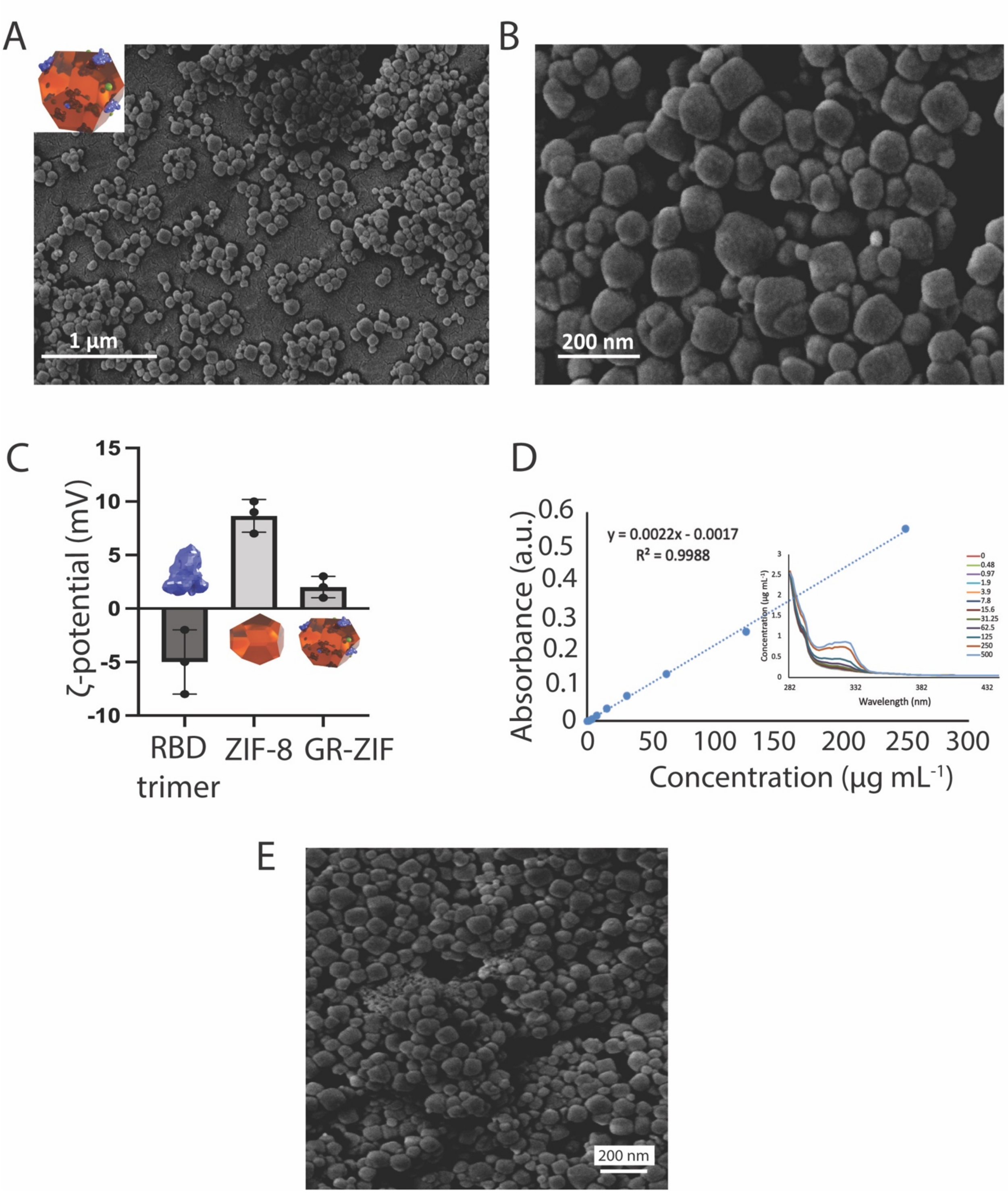

Fig. S1. Characterization of GR-ZIF. (**A, B**) SEM micrographs of GR-ZIF at low (**A**) and high magnification (**B**). (**C**) ζ-potential of RBD trimer, ZIF-8 and GR-ZIF, error bars are based on SD (n = 3). (**D**) Calibration plots of the absorbance as a function of Gdq concentrations, abs = 324 nm. (**E**) SEM micrographs of GR-ZIF in solution after 4h.

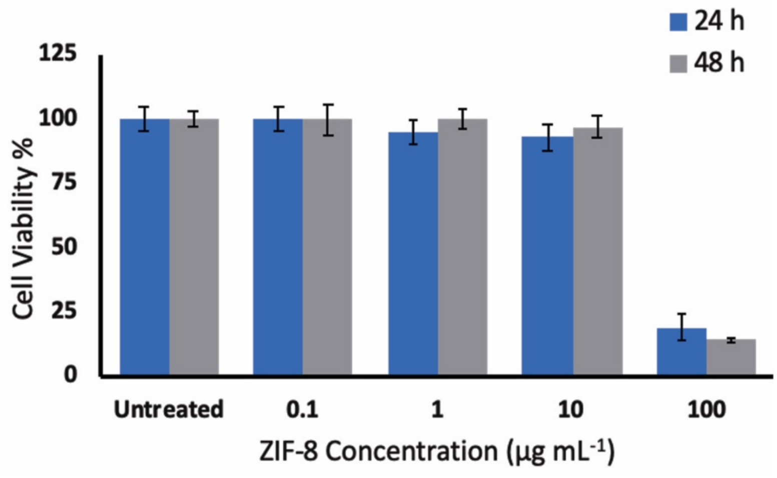

Figure S2. ZIF-8 *in vitro* biocompatibility. BMDCs were treated with different concentrations of B-ZIF for 24 h and 48 h to measure viability by CKK-8 assay.

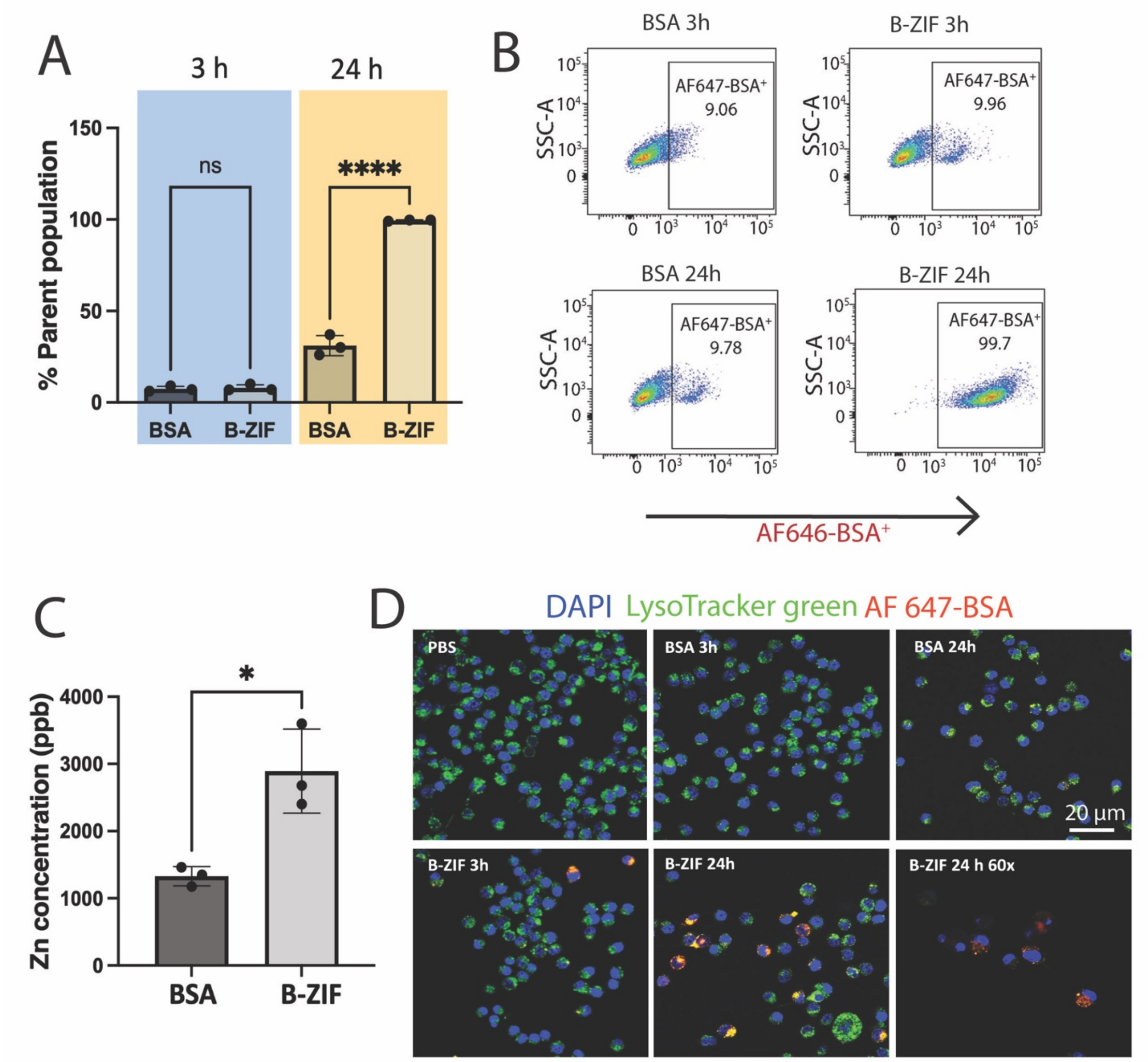

Figure S3. ZIF-8 promotes antigen uptake and presentation by DCs. (**A**, **B**) BMDCs were treated with soluble AF647-BSA or B-ZIF to assess antigen uptake and presentation by flow cytometry. (**C**) Amount of Zn^{2+} up taken by BMDCs 24 h post-incubation measured by ICP-MS. (**D**) Confocal laser scanning microscopy (CLSM) of AF647-BSA and B-ZIF following incubation with BMDCs for 3 h and 24 h. Lysotracker green (green) was used to stain the acidic organelles (endosomes) and nuclei were stained with Hoechst 33342. Scale bars = 20 μm.

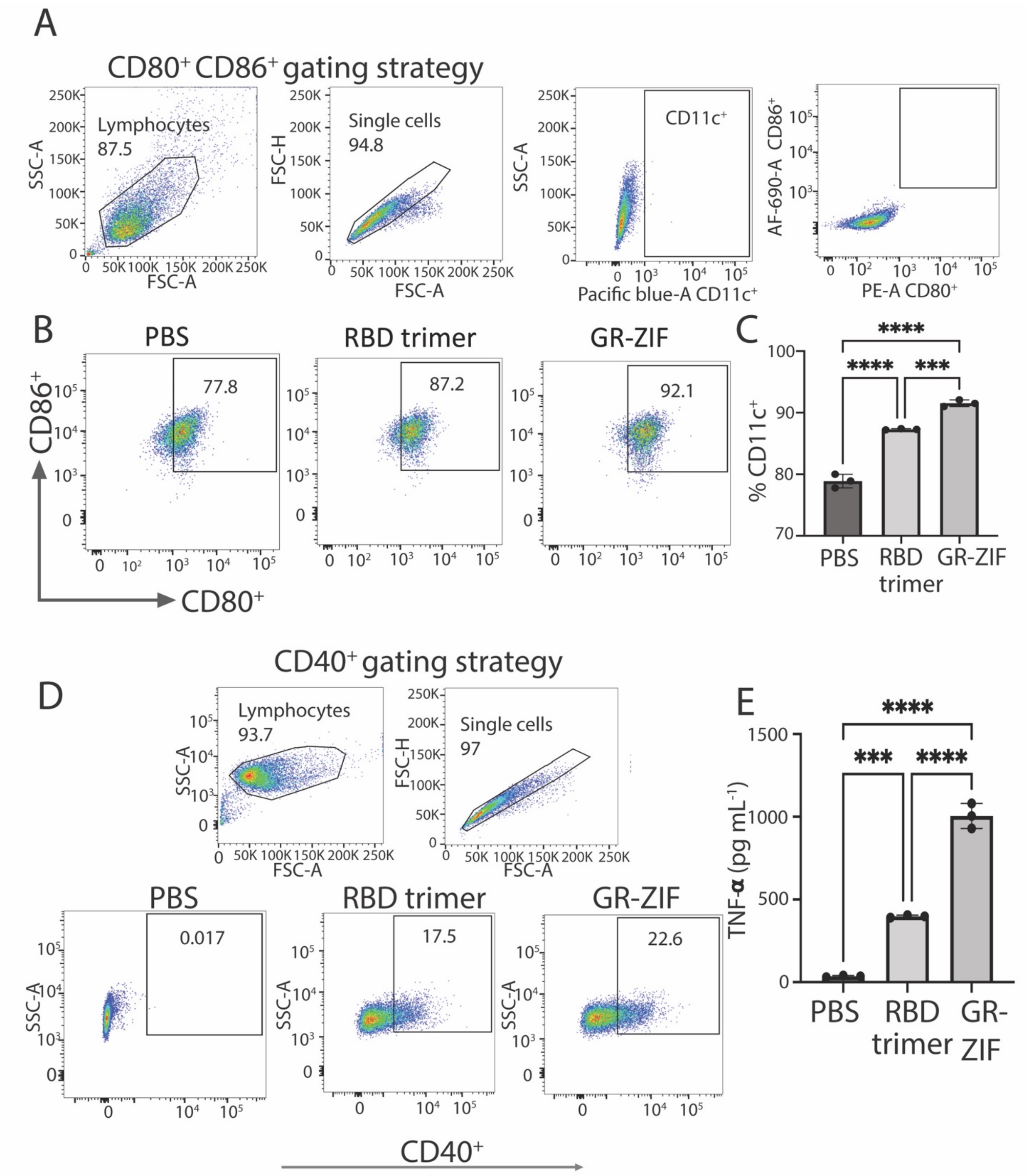

Figure S4. **GR-ZIF promotes the activation of DCs.** (**A**) Gating strategy of CD80⁺ and CD86⁺ DCs. (**B-D**) BMDCs were treated with soluble RBD trimer or GR-ZIF for 3 h or 24 h, followed by the measurement of CD80 and CD86 expression (**b, c**), CD40 (**D**) on BMDCs. (**E**) Quantitative analysis of TNF-**α** in the supernatant collected 48 h post-treatment measured by ELISA. Statistical significance was calculated by Student t-test: *$P < 0.05$, **$P < 0.01$, ***$P < 0.001$, and ****$P < 0.0001$. Data are shown as means ± SEM n=3.

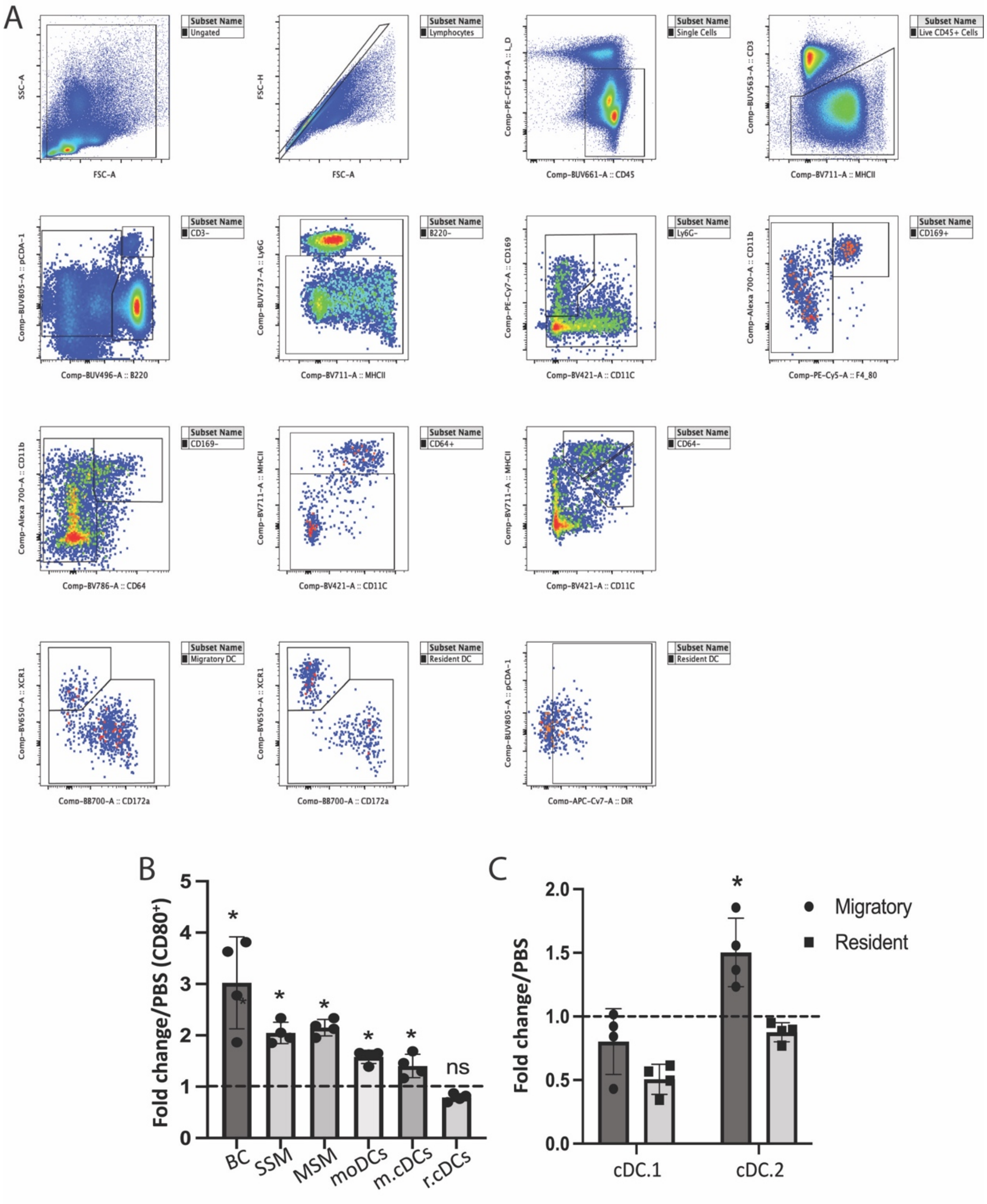

Figure S5. Flow cytometry analysis of D-ZIF in dLNs harvested 24 h post-injection. (**A**) After gating on lymphocytes, single cells, and live $CD45^{+}$ cells, draining lymph node populations of T cells, B cells, plasmacytoid DCs, neutrophils, subcapsular sinus macrophages (SSM), medullary sinus macrophages (MSM), resident and migratory cDC1s, resident and migratory cDC2s and monocyte-derived DCs (moDC) are identified. The third graph in the bottom row shows the gate used to determine the proportion of DiR^{+} cells in each of these populations. (**B, C**) Expression of activation marker CD80 on APCs (**B**) and cDC (**C**) subsets. Statistical significance was calculated by t-test or one-way ANOVA: **P*< 0.05, ***P*< 0.01, ****P*< 0.001, and *****P*< 0.0001. Data are shown as means ± SEM n=3 or 4.

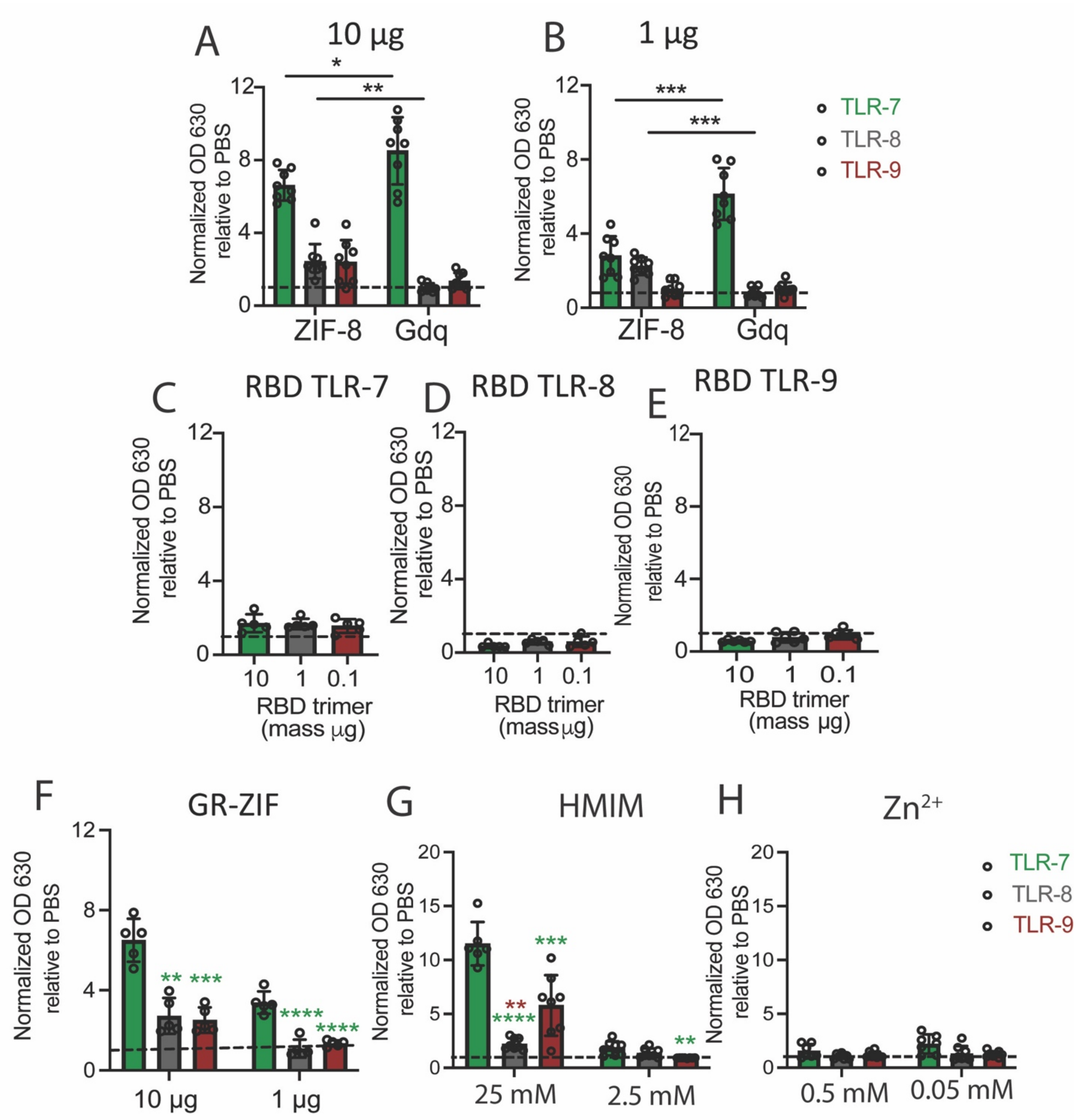

Figure S6. ZIF-8 activate TLR-7 at any given concentration, and TLR-8 and TLR-9 at high concentration. Activation of TLR-7, TLR-8 and TLR-9 in response to ZIF-8 and Gdq (**A, B**), RBD trimer (**C-E**), GR-ZIF (**F**), HMIM (**G**) and Zn^{2+} (**H**) in cultures of HEK-Blue TLR-7, TLR-8 and TLR-9 reporter cells following 24 h incubation.

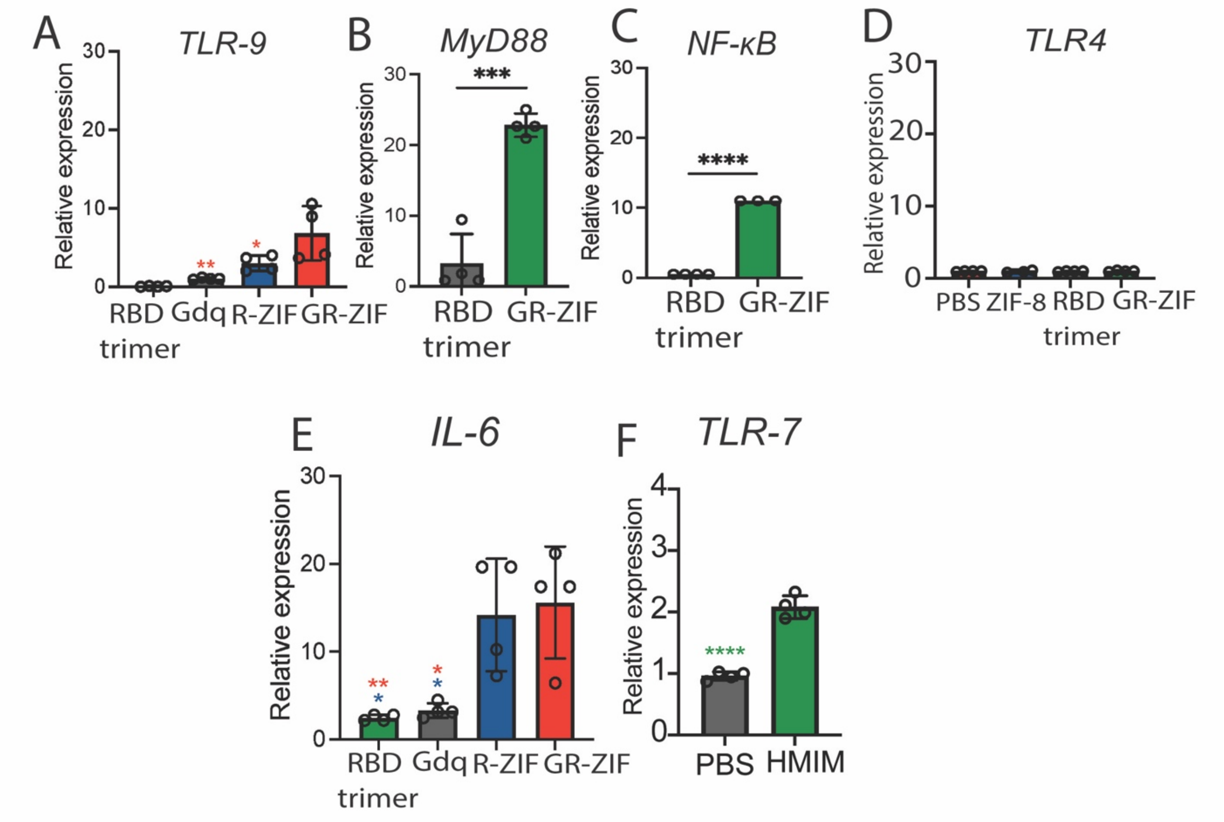

Figure S7. GR-ZIF induces the expression of TLR-7 and 9 but not TLR-4. C57BL/6 mice were intradermally injected with 3 µg of soluble RBD trimer, 0.8 µg Gdq and 0.9 µg of RBD trimer displayed on ZIF-8 with or without Gdq (**A-E**), or PBS and HMIM (f). dLNs were harvested 24 h post-injection to assess the relative expression of *TLR-9* (**A**), *MyD88* (**B**), *NF-kB* (**C**), *TLR-4* (**D**), *IL-6* (**E**) and *TLR-7* (**F**) by RT-qPCR.

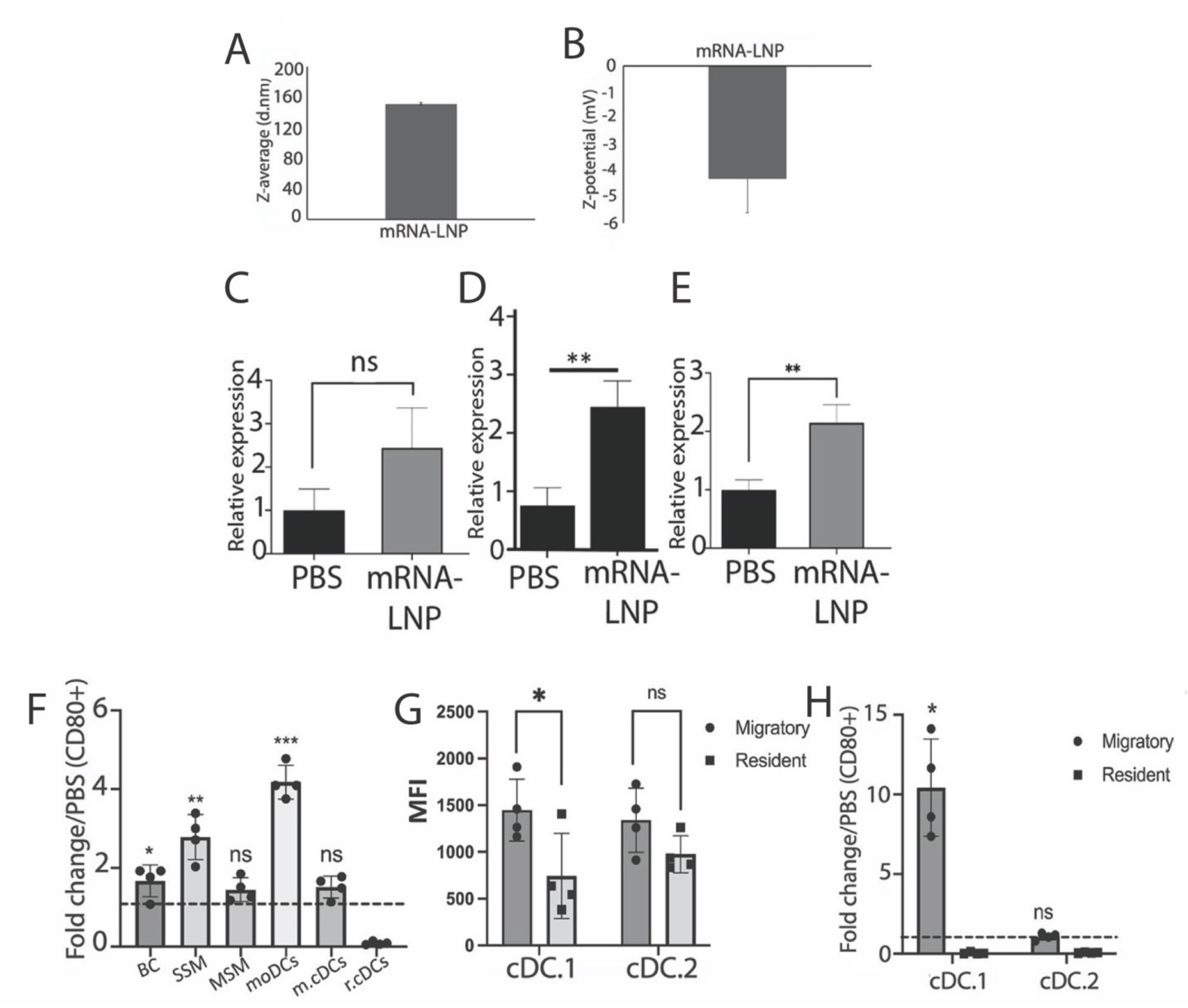

Figure S8. mRNA-LNP activates skin moDCs. (**A**)mRNA-LNP size as measured by dynamic light scattering (n = 3). (**B**) Zeta potential of mRNA-LNP. C57BL/6 mice were intradermally injected with mRNA-LNP, dLNs were isolated 24h post-immunization. (**C-E**) C57BL/6 mice were intradermally injected with mRNA-LNP, dLNs were harvested at week 5, a week after the booster dose. Relative expression of *TLR-7* (**C**), *TLR-9* (**D**) and *NF-kB* (**E**) in dLN of mRNA-LNP treated mice as measured by qPCR. (**F**) Flow cytometry analysis of dLN depicting the expression of activation marker CD80 on APCs. (**G**) mRNA-LNP distribution in cDC subsets given by MFI in which SSM and moDCs were highly associated with mRNA-LNP. (**H**) Expression of activation marker CD80 on cDC subsets. (**F**) Heat map illustrates results of APC polarization, in which moDC showed the highest activation levels. Statistical significance was calculated by t-test or one-way ANOVA: *P< 0.05, **P< 0.01, ***P< 0.001, and ****P< 0.0001. Data are shown as means ± SEM n=3 or 4.

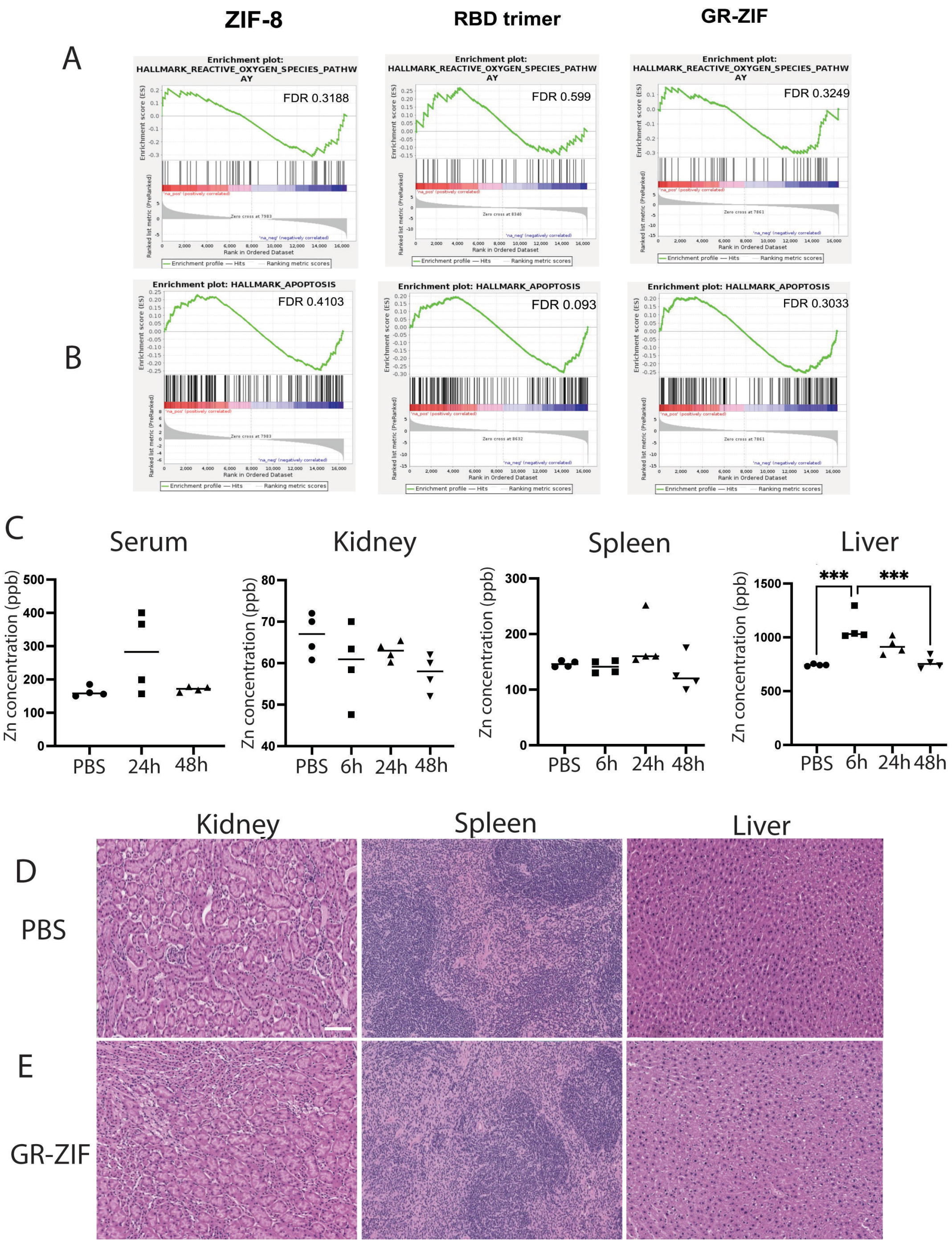

Figure S9. Biocompatibility and Safety of ZIF-8. (**A, B**) GSEA plots showing no enrichment of ROS- (**A**) and apoptosis- (**B**) related gene sets in dLN of ZIF-8 treated mice. The normalized enrichment scores (NES) and false discovery rate q value (FDR q) were indicated. (**C**) ICP-MS analysis of Zn in serum, kidneys, spleen and liver up to 48 h post-injection (n = 4 per group per time point). (**D, E**) Representative histology sections stained with H&E 24h after a single ID administration of PBS and GR-ZIF in kidney, spleen and liver. Statistical significance was calculated by Student's *t*-test: *$P < 0.05$, **$P < 0.01$, ***$P < 0.1$, and ****$P < 0.0001$. Data are shown as means ± SEM n=4.

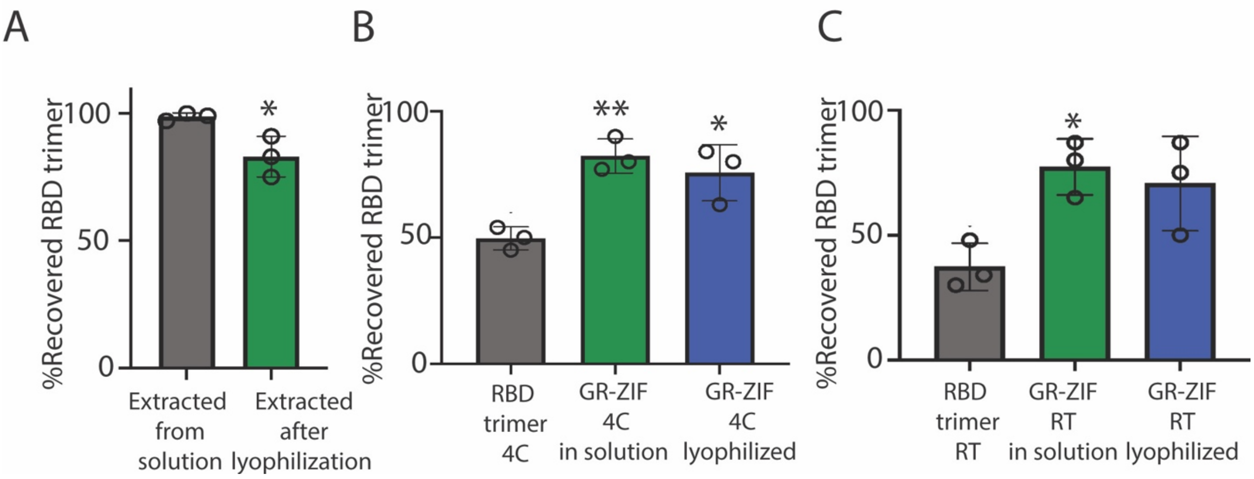

Figure S10. RBD trimer stability upon storage in different conditions. (A) RBD trimer stability after GR-ZIF lyophilization. **(B, C)** RBD trimer extracted from GR-ZIF after 60 days of storage **at** 4 °C **(B)** and room temperature **(C)**. Statistical significance was calculated by one-way ANOVA: *P < 0.05, **P < 0.01, ***P < 0.001, and ****P < 0.000 1. Data are shown as means ± SEM n=3.

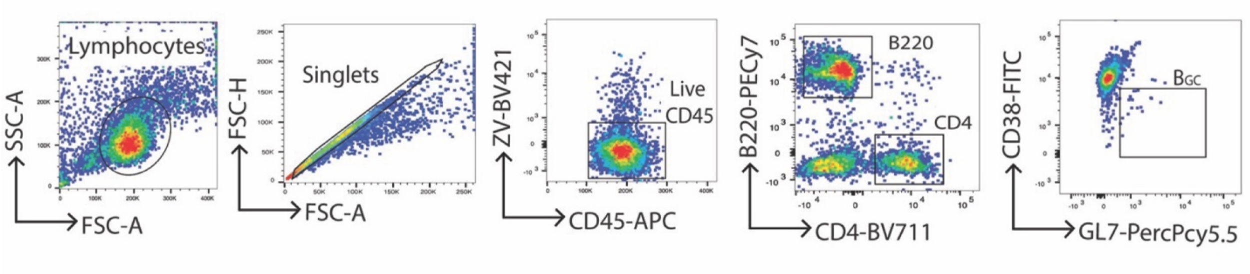

Figure S11. Gating strategies to identify B_{GC} in brachial LNs harvested 24 h post-injection. After gating on lymphocytes, single cells, and live $CD45^+$ cells were gated on $B220^+$, $CD4^-$, $CD38^{lo}$ and $GL7^{hi}$.

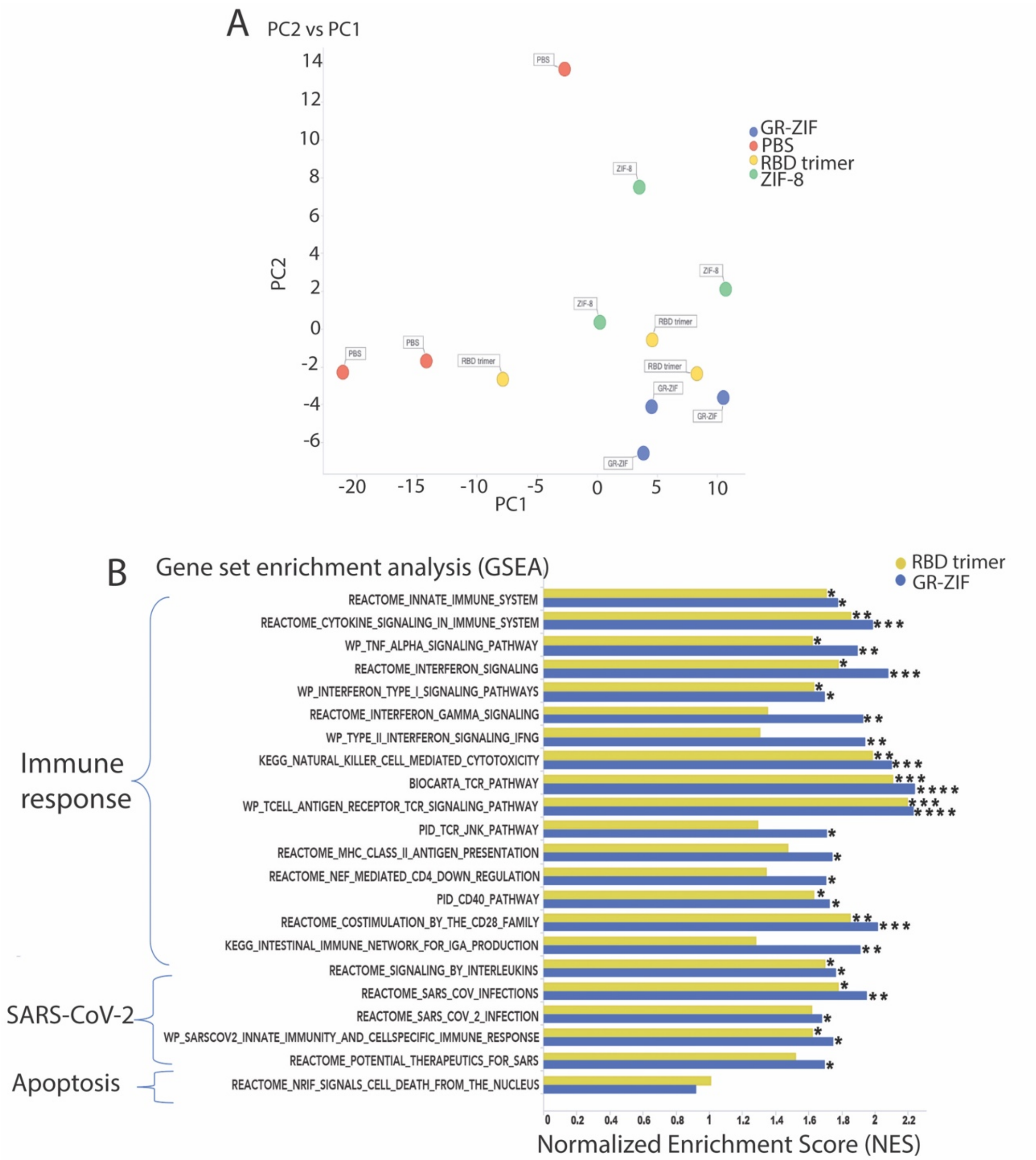

Figure S12. Gene set enrichment analysis (GSEA) showed significant upregulation of 17 pathways in dLNs of GR-ZIF immunized mice. (**A**) Principal component analysis (PCA) of gene expression following vaccination with PBS, RBD trimer, or GR-ZIF. (**B**) Gene ontology analysis of the differentially expressed genes in RBD trimer or GR-ZIF treated groups, which depicts the upregulation of 17 GR-ZIF pathways related to RBD trimer presentation on APC, T cell activation, and IFN-γ production. Distribution of the GO categories were assigned into three categories: immune response, SARS-CoV2 and cell death. The Wald statistic (log fold change divided by standard error) from DESeq2 differential expression analysis compares three replicates of each condition was used as the ranking metric in pre-ranked GSEA. This statistic orders genes from high in treated conditions (RBD trimer, GR-ZIF) to high in control. The magnitude of the normalized enrichment scores (NES) produced by GSEA indicates the degree of enrichment observed for each gene set.

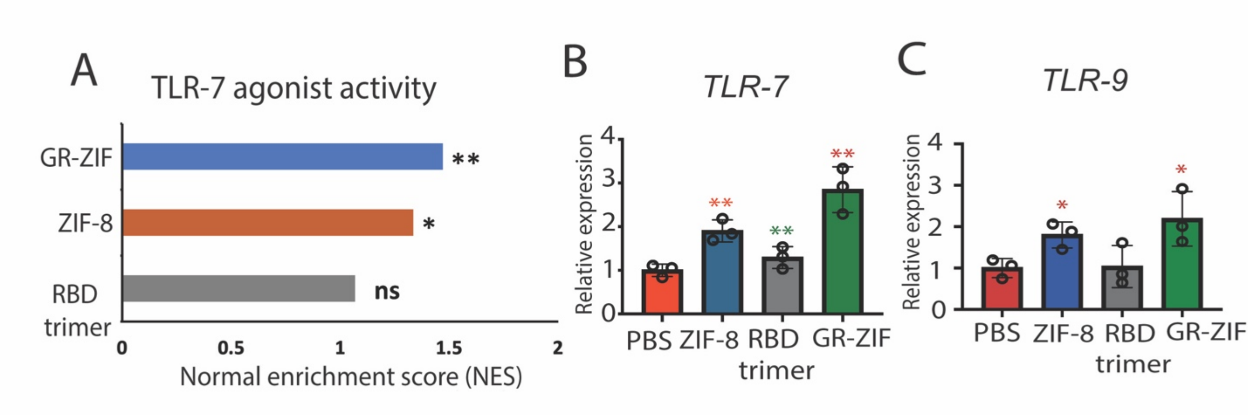

Figure S13. Gene expression analysis. (**A**) C57BL/6 mice were immunized intradermally with 3 µg of soluble RBD trimer, ZIF-8 or 0.9 µg of RBD trimer displayed on ZIF-8 with Gdq, and boosted on weeks 3 with the same formulations. One week after first booster dose, dLNs were harvested for RNA sequencing and RT-qPCR. KEGG enrichment focused on TLR-7 pathway, summarized based on the enrichment score. (**B, C**) Relative *TLR-7* and *TLR-9* expression in response to PBS, ZIF-8, RBD trimer and GR-ZIF validating RNA sequencing results. Statistical significance was calculated by Student's *t*-test: *$P < 0.05$, **$P < 0.01$, ***$P < 0.001$, and ****$P < 0.0001$. Data are shown as means ± SEM n=3.

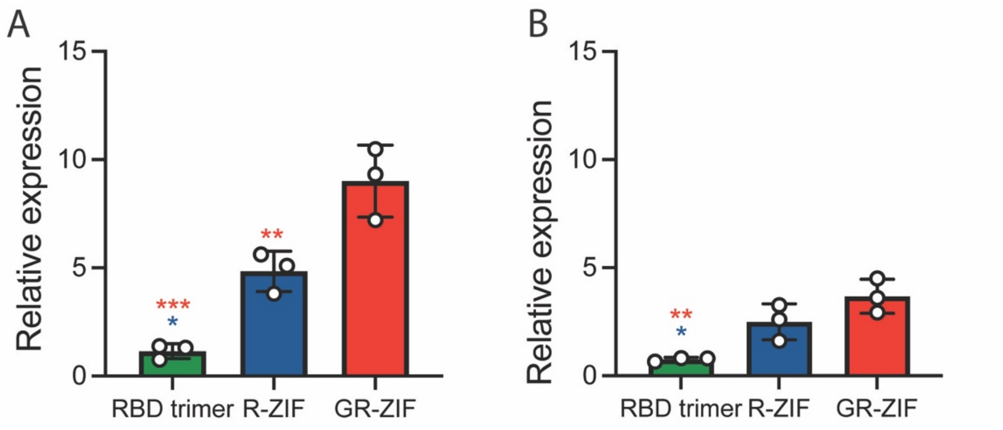

Figure S14. Compare the expression of *AICDA* and *TRAV1* in dLNs of C57BL/6 mice intradermally injected with RBD trimer, R-ZIF or GR-ZIF. dLNs were harvested at week 5, a week after the booster dose. Relative expression of *AICDA* (**A**) and *TRAV16* (**B**) in dLN as measured by RT-qPCR.

Table S1. Immunophenotypes of antigen-presenting cell populations identified in flow cytometric analysis of the draining brachial lymph node

T cells	CD3+
B cells	CD3- B220+
Plasmacytoid DCs	DCs: CD3- B220+ pCDA-1+
Subcapsular sinus macrophages (SSM)	CD3- B220- Ly-6G- CD11c- CD11b+ CD169+ F4/80-
Medullary sinus macrophages (MSM)	CD3- B220- Ly-6G- CD11c- CD11b+ CD169+ F4/80+
Inflammatory DCs	CD3- B220- Ly-6G- CD64+ CD11c+ MHCIIhi
Migratory cDCs	CD3- B220- Ly-6G- CD64- CD11c+ MHCIImid CCR7hi
Resident cDCs	CD3- B220- Ly-6G- CD64- CD11c+ MHCIIhi CCR7mid/lo
cDC1	XCR1+ migratory or resident cDCs
cDC2	CD172a+ migratory or resident cDCs

Table S2. PCR primer pairs

	Forward	Reverse
TLR-9	TATCCACCACCTGCACAACT	TTCAGCTCCTCCAGTGTACG
TLR-4	TTTATTCAGAGCCGTTGGTG	CAGAGGATTGTCCTCCCATT
TLR-3	TCACTTGCTCATTCTCCCTT	GACCTCTCCATTCCTGGC
TLR7	GATCGTGGACTGCACAGACAA	GGAATGCCCTCAGGGATTTC
MYD88	CCTTGATGACCCCCTAGGACA	CAGATAAAGGCATCGAAAAGTTCC
NF-kB	CTGGACATGGCTGCCAACT	CGGTTTCCCATTTAGTATGTCAAA
IL-6	CCAATTTCCAATGCTCTCCT	ACCACAGTGAGGAATGTCCA
IL-12p40	AGAGGAGGGGTGTAACCAG	CTGGTTACACCCCTCCTCT
β-Actin	TGACGGGGTTCACCCACACTGTGCC CATCTA	CTAGAAGCATTTGTGGTGGACGATGGAG GG
AICDA	CTGTGAAGACCGCAAGGCTGAG	AATTTTCATGTAGCCCTTCCCAGG
TRAV16	GGAAAATGCAACAGTGGGTC	GTGACAATGGACTGTGTGTATGAAACCG

REFERENCES AND NOTES

1. E. Caproni, E. Tritto, M. Cortese, A. Muzzi, F. Mosca, E. Monaci, B. Baudner, A. Seubert, E. De Gregorio, MF59 and Pam3CSK4 boost adaptive responses to influenza subunit vaccine through an IFN type I-independent mechanism of action. *J. Immunol.* **188**, 3088–3098 (2012).

2. H. Lal, A. L. Cunningham, O. Godeaux, R. Chlibek, J. Diez-Domingo, S. J. Hwang, M. J. Levin, J. E. McElhaney, A. Poder, J. Puig-Barbera, T. Vesikari, D. Watanabe, L. Weckx, T. Zahaf, T. C. Heineman, Efficacy of an adjuvanted herpes zoster subunit vaccine in older adults. *N. Engl. J. Med.* **372**, 2087–2096 (2015).

3. N. Wang, J. Shang, S. Jiang, L. Du, Subunit vaccines against emerging pathogenic human coronaviruses. *Front. Microbiol.* **11**, 298 (2020).

4. A. Bershteyn, M. C. Hanson, M. P. Crespo, J. J. Moon, A. V. Li, H. Suh, D. J. Irvine, Robust IgG responses to nanograms of antigen using a biomimetic lipid-coated particle vaccine. *J. Control. Release* **157**, 354–365 (2012).

5. C. Keech, G. Albert, I. Cho, A. Robertson, P. Reed, S. Neal, J. S. Plested, M. Zhu, S. Cloney-Clark, H. Zhou, G. Smith, N. Patel, M. B. Frieman, R. E. Haupt, J. Logue, M. McGrath, S. Weston, P. A. Piedra, C. Desai, K. Callahan, M. Lewis, P. Price-Abbott, N. Formica, V. Shinde, L. Fries, J. D. Lickliter, P. Griffin, B. Wilkinson, G. M. Glenn, Phase 1-2 trial of a SARS-CoV-2 recombinant spike protein nanoparticle vaccine. *N. Engl. J. Med.* **383**, 2320–2332 (2020).

6. S. G. Reed, M. T. Orr, C. B. Fox, Key roles of adjuvants in modern vaccines. *Nat. Med.* **19**, 1597–1608 (2013).

7. B. Pulendran, P. S. Arunachalam, D. T. O'Hagan, Emerging concepts in the science of vaccine adjuvants. *Nat. Rev. Drug Discov.* **20**, 454–475 (2021).

8. R. Medzhitov, Toll-like receptors and innate immunity. *Nat. Rev. Immunol.* **1**, 135–145 (2001).

9. G. P. Amarante-Mendes, S. Adjemian, L. M. Branco, L. C. Zanetti, R. Weinlich, K. R. Bortoluci, Pattern recognition receptors and the host cell death molecular machinery. *Front. Immunol.* **9**, 2379 (2018).

10. S. Akira, S. Uematsu, O. Takeuchi, Pathogen recognition and innate immunity. *Cell* **124**, 783–801 (2006).

11. S. P. Kasturi, I. Skountzou, R. A. Albrecht, D. Koutsonanos, T. Hua, H. I. Nakaya, R. Ravindran, S. Stewart, M. Alam, M. Kwissa, F. Villinger, N. Murthy, J. Steel, J. Jacob, R. J. Hogan, A. Garcia-Sastre, R. Compans, B. Pulendran, Programming the magnitude and persistence of antibody responses with innate immunity. *Nature* **470**, 543–547 (2011).

12. G. M. Lynn, C. Sedlik, F. Baharom, Y. Zhu,R. A. Ramirez-Valdez, V. L. Coble, K. Tobin, S. R. Nichols, Y. Itzkowitz, N. Zaidi, J. M. Gammon, N. J. Blobel, J. Denizeau, P. de la Rochere, B. J. Francica, B. Decker, M. Maciejewski, J. Cheung, H. Yamane,M. G. Smelkinson, J. R. Francica, R. Laga,J. D. Bernstock, L. W. Seymour, C. G. Drake, C. M. Jewell, O. Lantz, E. Piaggio, A. S. Ishizuka, R. A. Seder, Peptide-TLR-7/8a conjugate vaccines chemically programmed for nanoparticle self-assembly enhance CD8 T-cell immunity to tumor antigens. *Nat. Biotechnol.* **38**, 320–332 (2020).

13. B. Sun, T. Xia, Nanomaterial-based vaccine adjuvants. *J. Mater. Chem. B* **4**, 5496–5509 (2016).

14. G. M. Lynn, R. Laga, P. A. Darrah, A. S. Ishizuka, A. J. Balaci, A. E. Dulcey, M. Pechar, R. Pola, M. Y. Gerner, A. Yamamoto, C. R. Buechler, K. M. Quinn, M. G. Smelkinson, O. Vanek, R. Cawood, T. Hills, O. Vasalatiy, K. Kastenmuller, J. R. Francica, L. Stutts, J. K. Tom, K. A. Ryu, A. P. Esser-Kahn, T. Etrych, K. D. Fisher, L. W. Seymour, R. A. Seder, In vivo characterization of the physicochemical properties of polymer-linked TLR agonists that enhance vaccine immunogenicity. *Nat. Biotechnol.* **33**, 1201–1210 (2015).

15. M. Silva, Y. Kato, M. B. Melo, I. Phung, B. L. Freeman, Z. Li, K. Roh, J. W. Van Wijnbergen, H. Watkins, C. A. Enemuo, B. L. Hartwell, J. Y. H. Chang, S. Xiao, K. A. Rodrigues, K. M. Cirelli, N. Li, S. Haupt, A. Aung, B. Cossette, W. Abraham, S. Kataria, R. Bastidas,J. Bhiman, C. Linde, N. I. Bloom, B. Groschel, E. Georgeson, N. Phelps,A. Thomas, J. Bals, D. G.

Carnathan, D. Lingwood, D. R. Burton, G. Alter, T. P. Padera, A. M. Belcher, W. R. Schief, G. Silvestri, R. M. Ruprecht, S. Crotty, Irvine, D. J, A particulate saponin/TLR agonist vaccine adjuvant alters lymph flow and modulates adaptive immunity. *Sci. Immunol.* **6**, eabf1152 (2021).

16. C. G. Kim, Y. C. Kye, C. H. Yun, The role of nanovaccine in cross-presentation of antigen-presenting cells for the activation of $CD8^+$ T cell responses. *Pharmaceutics* **11**, 612 (2019).

17. R. S. Oakes, L. H. Tostanoski, S. M. Kapnick, E. Froimchuk, S. K. Black, X. Zeng, C. M. Jewell, Exploiting rational assembly to map distinct roles of regulatory cues during autoimmune therapy. *ACS Nano* **15**, 4305–4320 (2021).

18. M. L. Bookstaver, Q. Zeng, R. S. Oakes, S. M. Kapnick, V. Saxena, C. Edwards, N. Venkataraman, S. K. Black, X. Zeng, E. Froimchuk, T. Gebhardt, J. S. Bromberg, C. M. Jewell, Self-assembly of immune signals to program innate immunity through rational adjuvant design. *Adv. Sci.* **10**, e2202393 (2023).

19. M. T. Abrams, M. L. Koser, J. Seitzer, S. C. Williams, M. A. DiPietro, W. Wang, A. W. Shaw, X. Mao, V. Jadhav, J. P. Davide, P. A. Burke, A. B. Sachs, S. M. Stirdivant, L. Sepp-Lorenzino, Evaluation of efficacy, biodistribution, and inflammation for a potent siRNA nanoparticle: Effect of dexamethasone co-treatment. *Mol. Ther.* **18**, 171–180 (2010).

20. K. Liang, R. Ricco, C. M. Doherty, M. J. Styles, S. Bell, N. Kirby, S. Mudie, D. Haylock, A. J. Hill, C. J. Doonan, P. Falcaro, Biomimetic mineralization of metal–organic frameworks as protective coatings for biomacromolecules. *Nat. Commun.* **6**, 7240 (2015).

21. C. Wang, G. Sudlow, Z. Wang, S. Cao, Q. Jiang, A. Neiner,J. J Morrissey, E. D. Kharasch, S. Achilefu, S. Singamaneni, Metal-organic framework encapsulation preserves the bioactivity of protein therapeutics. *Adv. Healthc. Mater.* **7**, e1800950 (2018).

22. S. K. Alsaiari, S. Patil, M. Alyami, K. O. Alamoudi, F. A. Aleisa, J. S. Merzaban, M. Li, N. M. Khashab, Endosomal escape and delivery of CRISPR/Cas9 genome editing machinery enabled by nanoscale zeolitic imidazolate framework. *J. Am. Chem. Soc.* **140**, 143–146 (2018).

23. Y. W. Zhang, F. Wang, E. Ju, Z. Liu, Z. Chen, J. Ren, X. Qu, Metal-organic-framework-based vaccine platforms for enhanced systemic immune and memory response. *Adv. Funct. Mater.* **26**, 6454–6461 (2016).

24. G. Zhang, X. Fu, H. Sun, P. Zhang, S. Zhai, J. Hao, J. Cui, J. M. Hu, Poly(ethylene glycol)-mediated assembly of vaccine particles to improve stability and immunogenicity. *ACS Appl. Mater. Interfaces* **13**, 13978–13989 (2021).

25. L. Wang, G. Zhang, Y. Sun, Z. Wu, C. Ren, Z. Zhang, X. Peng, Y. Zhang, Y. Zhao, C. Li, L. Gao, X. Liang, H. Sun, J. Cui, C. Ma, Enhanced delivery of TLR7/8 agonists by metal-organic frameworks for hepatitis B virus cure. *ACS Appl. Mater. Interfaces* **14**, 46176–46187 (2022).

26. Y. Yang, A. Csakai, S. Jiang, C. Smith, H. Tanji, J. Huang, T. Jones, K. Sakaniwa, L. Broadwell, C. Shi, S. Soti, U. Ohto, Y. Fang, S. Shen, F. Deng, T. Shimizu, H. Yin, Tetrasubstituted imidazoles as incognito Toll-like receptor 8 a(nta)gonists. *Nat. Commun.* **12**, 4351 (2021).

27. M. Beesu, G. Caruso, A. C. Salyer, N. M. Shukla, K. K. Khetani, L. J. Smith, L. M. Fox, H. Tanji, U. Ohto, T. Shimizu, S. A. David, Identification of a human Toll-like receptor (TLR) 8-specific agonist and a functional pan-TLR inhibitor in 2-aminoimidazoles. *J. Med. Chem.* **59**, 3311–3330 (2016).

28. G. V. Reynoso, A. S. Weisberg, J. P. Shannon, D. T. McManus, L. Shores, J. L. Americo, R. V. Stan, J. W. Yewdell, H. D. Hickman, Lymph node conduits transport virions for rapid T cell activation. *Nat. Immunol.* **20**, 602–612 (2019).

29. S. Calabro, M. Tortoli, B. C. Baudner, A. Pacitto, M. Cortese,D. T. O'Hagan, E. De Gregorio, A. Seubert, A. Wack, Vaccine adjuvants alum and MF59 induce rapid recruitment of neutrophils and monocytes that participate in antigen transport to draining lymph nodes. *Vaccine* **29**, 1812–1823 (2011).

30. V. Manolova, A. Flace, M. Bauer, K. Schwarz, P. Saudan, M. F. Bachmann, Nanoparticles target distinct dendritic cell populations according to their size. *Eur. J. Immunol.* **38**, 1404–1413 (2008).

31. N. Bhardwaj, A. Bender, N. Gonzalez, L. K. Bui, M. C. Garrett, R. M. Steinman, Influenza virus-infected dendritic cells stimulate strong proliferative and cytolytic responses from human CD8+ T cells. *J. Clin. Invest.* **94**, 797–807 (1994).

32. A. S. McWilliam, A. M. Marsh, P. G. Holt, Inflammatory infiltration of the upper airway epithelium during Sendai virus infection: Involvement of epithelial dendritic cells. *J. Virol.* **71**, 226–236 (1997).

33. A. L. Musumeci, K. Winheim, E. Krug, What makes a pDC: Recent advances in understanding plasmacytoid DC development and heterogeneity. *Front. Immunol.* **10**, 1222 (2019).

34. F. Ma, J. Zhang, J. Zhang, C. Zhang, The TLR7 agonists imiquimod and gardiquimod improve DC-based immunotherapy for melanoma in mice. *Cell. Mol. Immunol.* **7**, 381–388 (2010).

35. S. Bhagchandani, J. A. Johnson, D. J. Irvine, Evolution of Toll-like receptor 7/8 agonist therapeutics and their delivery approaches: From antiviral formulations to vaccine adjuvants. *Adv. Drug Deliv. Rev.* **175**, 113803 (2021).

36. T. Worbs, S. I. Hammerschmidt, R. Forster, Dendritic cell migration in health and disease. *Nat. Rev. Immunol.* **17**, 30–48 (2017).

37. A. Mildner, S. Jung, Development and function of dendritic cell subsets. *Immunity* **40**, 642–656 (2014).

38. T. H. Mogensen, Pathogen recognition and inflammatory signaling in innate immune defenses. *Clin. Microbiol. Rev.* **22**, 240–273 (2009).

39. M. Rescigno, M. Martino, C. L. Sutherland, M. R. Gold, P. Ricciardi-Castagnoli, Dendritic cell survival and maturation are regulated by different signaling pathways. *J. Exp. Med.* **188**, 2175–2180 (1998).

40. C. L. Doxsee, T. R. Riter, M. J. Reiter, S. J. Gibson, J. P. Vasilakos, R. M. Kedl, The immune response modifier and Toll-like receptor 7 agonist S-27609 selectively induces IL-12 and TNF-α production in $CD11c^{+}CD11b^{+}CD8^{-}$ dendritic cells. *J. Immunol.* **171**, 1156–1163 (2003).

41. A. Subramanian, P. Tamayo, V. K. Mootha, S. Mukherjee, B. L. Ebert, M. A. Gillette, A. Paulovich, S. L. Pomeroy, T. R. Golub, E. S. Lander, J. P. Mesirov, Gene set enrichment analysis: A knowledge-based approach for interpreting genome-wide expression profiles. *Proc. Natl. Acad. Sci. U.S.A.* **102**, 15545–15550 (2005).

42. S. R. Park, Activation-induced cytidine deaminase in B cell immunity and cancers. *Immune Netw.* **12**, 230–239 (2012).

43. I. Romero-Camarero, X. Jiang, Y. Natkunam, X. Lu, C. Vicente-Duenas, I. Gonzalez-Herrero, T. Flores, J. L. Garcia, G. McNamara, C. Kunder, S. Zhao, V. Segura, L. Fontan, J. A. Martinez-Climent, F. J. Garcia-Criado, J. D. Theis, A. Dogan, E. Campos-Sanchez, M. R. Green, A. A. Alizadeh, C. Cobaleda, I. Sanchez-Garcia, I. S. Lossos, Germinal centre protein HGAL promotes lymphoid hyperplasia and amyloidosis via BCR-mediated Syk activation. *Nat. Commun.* **4**, 1338 (2013).

44. M. S. Mulligan, A. B. Lentsch, M. Huber-Lang, R. F. Guo, V. Sarma, C. D. Wright, T. R. Ulich, P. A. Ward, Anti-inflammatory effects of mutant forms of secretory leukocyte protease inhibitor. *Am. J. Pathol.* **156**, 1033–1039 (2000).

45. R. J. Brownlie, R. Zamoyska, T cell receptor signalling networks: Branched, diversified and bounded. *Nat. Rev. Immunol.* **13**, 257–269 (2013).

46. J. Rossjohn, S. Gras, J. J. Miles, S. J. Turner, D. I. Godfrey, J. McCluskey, T cell antigen receptor recognition of antigen-presenting molecules. *Annu. Rev. Immunol.* **33**, 169–200 (2015).

47. S. Kumar, R. Sunagar, E. Gosselin, Bacterial protein Toll-like-receptor agonists: A novel perspective on vaccine adjuvants. *Front. Immunol.* **10**, 1144 (2019).

48. M. J. McCluskie, J. L. Cartier, A. J. Patrick, D. Sajic, R. D. Weeratna, K. L. Rosenthal, H. L. Davis, Treatment of intravaginal HSV-2 infection in mice: A comparison of CpG oligodeoxynucleotides and resiquimod (R-848). *Antiviral Res.* **69**, 77–85 (2006).

49. S. Jeong, Y. Choi, K. Kim, Engineering therapeutic strategies in cancer immunotherapy via exogenous delivery of Toll-like receptor agonists. *Pharmaceutics* **13,** 1374 (2021).

50. N. Baxan, A. Papanikolaou, I. Salles-Crawley,R. Chowdhury, O. Dubois, J. Branca, M. G. Hasham, N. Rosenthal, S. K. Prasad, L. Zhao, S. E. Harding, S. Sattler, Characterization of acute TLR-7 agonist-induced hemorrhagic myocarditis in mice by multi-parametric quantitative cardiac MRI. *Dis. Model. Mech.* **12,** dmm040725 (2019).

51. J. P. Vasilakos, M. A. Tomai. The use of Toll-like receptor 7/8 agonists as vaccine adjuvants. *Expert Rev. Vaccines* **12**, 809–819 (2013).

52. Q. Yin, W. Luo, V. Mallajosyula, Y. Bo, J. Guo, J. Xie, M. Sun, R. Verma, C. Li, C. M. Constantz, L. E. Wagar,J. Li, E. Sola, N. Gupta, C. Wang, O. Kask, X. Chen, X. Yuan, N. C. Wu, J. Rao, Y. H. Chien, J. Cheng, B. Pulendran, M. M. Davis, A TLR7-nanoparticle adjuvant promotes a broad immune response against heterologous strains of influenza and SARS-CoV-2. *Nat. Mater.* **22**, 380–390 (2023).

53. M. J. Mitchell, M. M. Billingsley, R. M. Haley, M. E. Wechsler, N. A. Peppas, R. Langer, Engineering precision nanoparticles for drug delivery. *Nat. Rev. Drug Discov.* **20**, 101–124 (2021).

54. N. G. Lamson, A. Berger, K. C. Fein, K. A. Whitehead. Anionic nanoparticles enable the oral delivery of proteins by enhancing intestinal permeability. *Nat. Biomed. Eng.* **4**, 84–96 (2020).

55. A. T. DiPiazza, J. P. Hill, B. S. Graham, T. J. Ruckwardt. OMIP-061: 20-color flow cytometry panel for high-dimensional characterization of murine antigen-presenting cells. *Cytometry A* **95**, 1226–1230 (2019).

56. R. Patro, G. Duggal, M. I. Love, R. A. Irizarry, C. Kingsford. Salmon provides fast and bias-aware quantification of transcript expression. *Nat. Methods* **14**, 417–419 (2017).

57. C. Soneson, M. I. Love, M. D. Robinson. Differential analyses for RNA-seq: Transcript level estimates improve gene-level inferences. *F1000Res* **4**, 1521 (2015).

58. R. C. Team. *R: A Language and Environment for Statistical Computing*, 2021).

59. M. I. Love, W. Huber, S. Anders. Moderated estimation of fold change and dispersion for RNA-seq data with DESeq2. *Genome Biol.* **15**, 550 (2014).

60. S. Anders, W. Huber. Differential expression analysis for sequence count data. *Genome Biol.* **11**, R106 (2010).

61. A. Zhu, J. G. Ibrahim, M. I. Love. Heavy-tailed prior distributions for sequence count data: Removing the noise and preserving large differences. *Bioinformatics* **35**, 2084–2092 (2019).

62. V. K. Mootha, C. M. Lindgren, K. F. Eriksson, A. Subramanian, S. Sihag, J. Lehar, P. Puigserver, E. Carlsson, M. Ridderstrale, E. Laurila, N. Houstis, M. J. Daly, N. Patterson, J. P. Mesirov, T. R. Golub, P. Tamayo, B. Spiegelman, E. S. Lander, J. N. Hirschhorn, D. Altshuler, L. C. Groop, PGC-1α-responsive genes involved in oxidative phosphorylation are coordinately downregulated in human diabetes. *Nat. Genet.* **34**, 267–273 (2003).

63. C. B. Maisonneuve, S. Philpott, D. J. De Gregorio, E., Unleashing the potential of NOD- and Toll-like agonists as vaccine adjuvants. *Proc. Natl. Acad. Sci. U.S.A.* **111**, (2014), 12294, 12299.

64. E. J. Hennessy, A. E. Parker, L. A. O'Neill, Targeting Toll-like receptors: Emerging therapeutics? *Nat. Rev. Drug Discov.* **9**, 293–307 (2010).

65. P. Chen, M. He, B. Chen, B. Hu, Size- and dose-dependent cytotoxicity of ZIF-8 based on single cell analysis. *Ecotoxicol. Environ. Saf.* **205**, 111110 (2020).

66. M. W. Hoopa, C. Riccòc, R. Mushtaqa, F. Terzopouloua, A. Chena, X-Z. deMellob, A. Doonand, C. Falcaroc, P. Nelsona, B. Puigmartí-Luisb, J. Panéa, S. Biocompatibility characteristics of the metal organic framework ZIF-8 for therapeutical applications *Mater. Today* **11**, 13–21 (2018).

67. D. J. A. Crommelin, T. J. Anchordoquy, D. B. Volkin, W. Jiskoot, E. Mastrobattista, Addressing the cold reality of mRNA vaccine stability. *J. Pharm. Sci.* **110**, 997–1001 (2021).

68. N. Dumpa, K. Goel, Y. Guo, H. McFall, A. R. Pillai, A. Shukla, M. A. Repka, S. N. Murthy, Stability of vaccines. *AAPS PharmSciTech* **20**, 42 (2019).

69. O. S. Kumru, S. B. Joshi, D. E. Smith, C. R. Middaugh, T. Prusik, D. B. Volkin, Vaccine instability in the cold chain: Mechanisms, analysis and formulation strategies. *Biologicals* **42**, 237–259 (2014).
